# Supplementary figures and images for: Muscleblind-1 interacts with tubulin mRNAs to regulate the microtubule cytoskeleton in C. elegans mechanosensory neurons
Source: PLoS Genet. 2023 Aug 21;19(8):e1010885. doi: 10.1371/journal.pgen.1010885 (PMC10470942; doi:10.1371/journal.pgen.1010885)

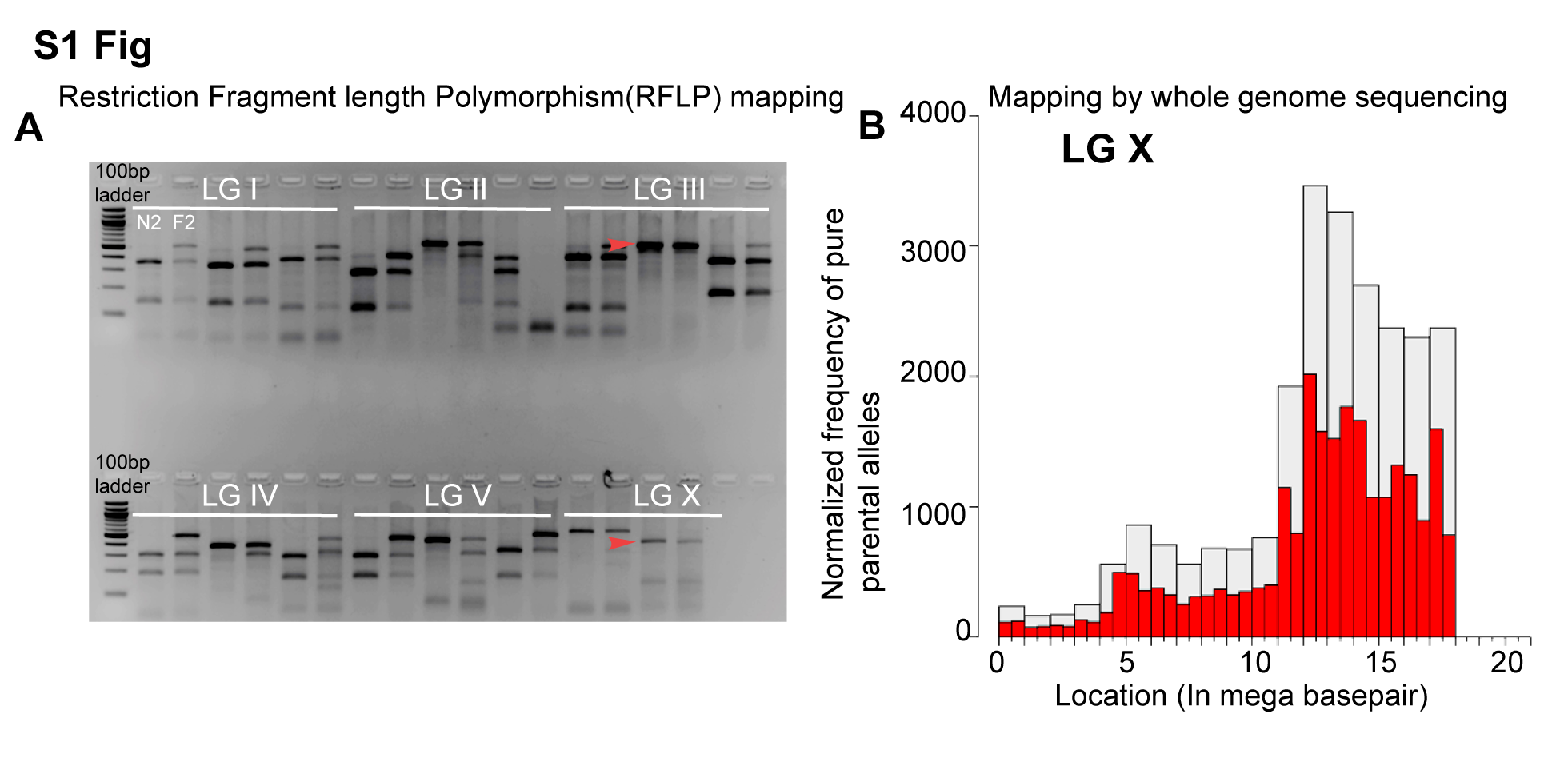

Supplement: S1 Fig — (A) The Gel picture showing the result of Restriction Fragment Length Polymorphism (RFLP) mapping of ju1128 mutation. The 100-base pair ladder was used as a marker. In the gel picture, mutation showed linkage on two chromosomes, one on the third chromosome, which is because of klp-7(tm2143) (marked with red arrowhead) and another on the X chromosome (marked with red arrowhead). (B) Frequency plot of X-chromosome for mapping of the ju1128 mutation from the whole genome sequencing data using the method described by Minevich et al. (2012), with chromosome position (in megabases/Mb) plotted against the Normalized frequency of pure parental alleles. It peaks at the genomic position linked to the ju1128 mutation. (TIF) [file pgen.1010885.s001.tif]

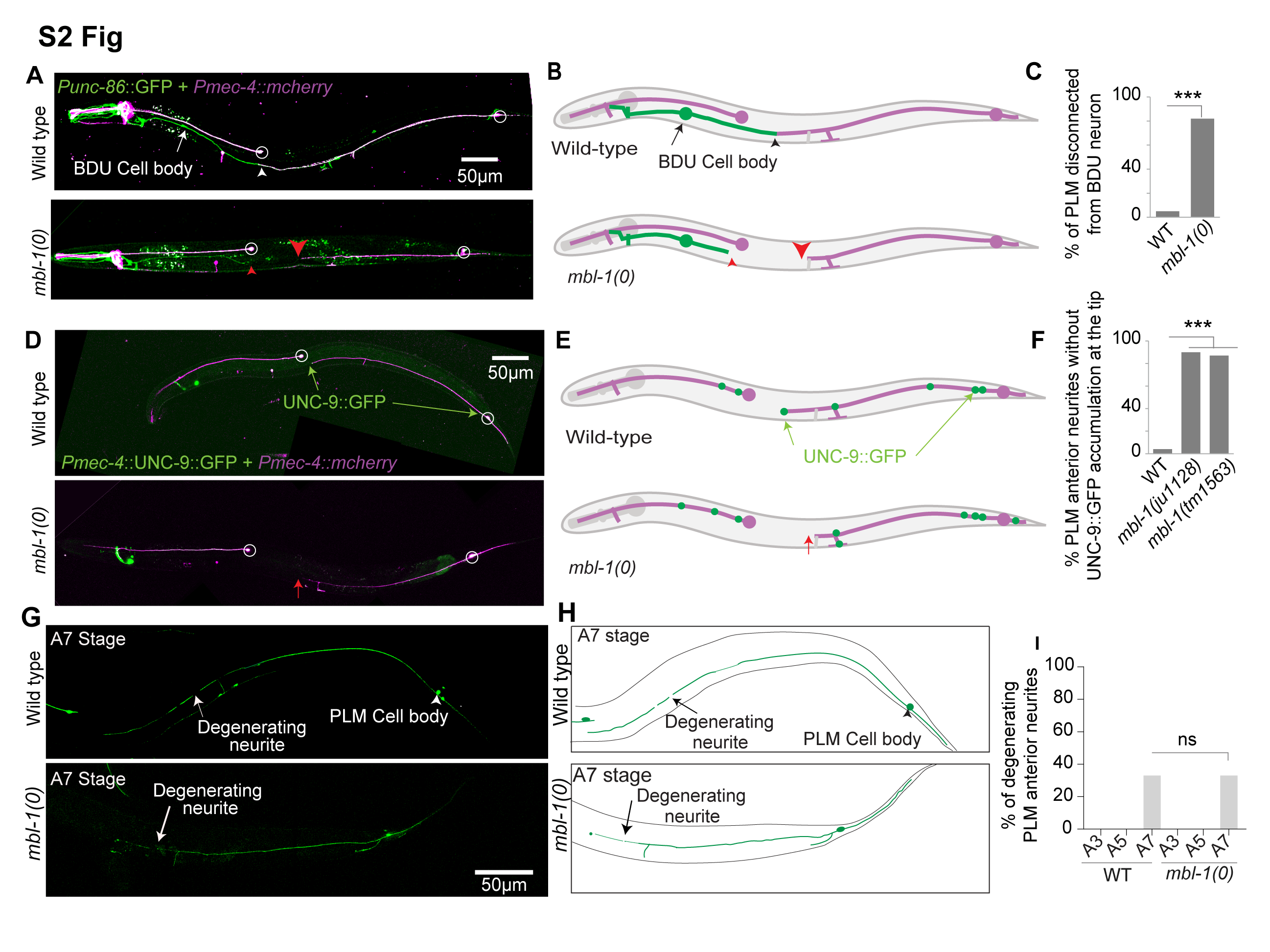

Supplement: S2 Fig — (A) Representative confocal images and (B) illustration of touch neurons (ALM and PLM) and BDU neurons in both wild-type and mbl-1(0) at the L4 stage. BDU neurons were visualized using Punc-86::GFP (kyIs262) and for visualization of touch neurons, Pmec-4::mCherry (tbIs222) and Punc-86::GFP (kyIs262) transgenes were used. The presence of physical contact between the PLM anterior neurite and BDU neuron is shown by the white arrowhead in the wild-type background which is lost in mbl-1(0) shown by the red arrowhead. (C) Quantification of the defect as shown in the image (A). N = 3 independent replicates, n (number of worms) = 25–32. (D-E) Representative confocal images (D) and schematics (E) of the gap junction synapse labeled with UNC-9::GFP(shrEx434) in the touch neurons, in the wild-type and mbl-1(0) backgrounds. The green arrows show the localization of UNC-9::GFP in the wild-type background, whereas in the mbl-1(0) the red arrow is pointed at the tip of PLM anterior neurite missing UNC-9::GFP localization. (F) Quantification of the percentage of defect shown in the image in panel D. N = 3–4 independent replicates, n (number of worms) = 30–35. (G-I) Representative confocal images (G) and illustration (H) of PLM neuron in the wild-type (muIs32) and mbl-1(tm1563); muIs32 backgrounds at A7 (seven-day adult) stage. White arrows are showing the visible gaps in the anterior neurite indicating degeneration. (I) Histogram showing the quantification of degeneration of PLM neurons at A3 (three-day adult stage), A5 (five-day adult stage), and A7 in wild-type and mbl-1(tm1563) background. N = 3 independent replicates, n (number of worms) = 16–26. For C, and F***P <0.001; Fisher’s exact test. ns, not significant. (TIF) [file pgen.1010885.s002.tif]

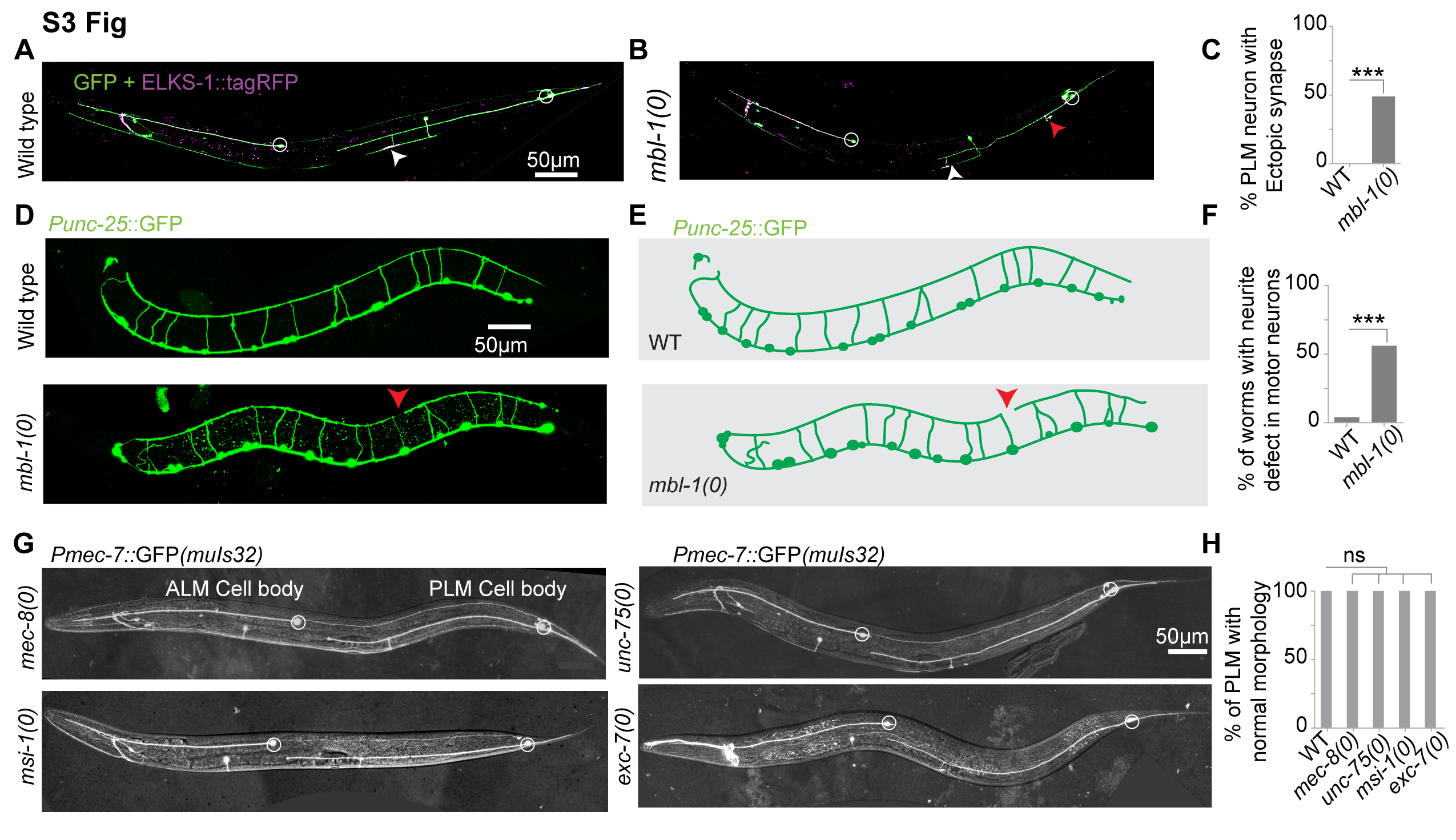

Supplement: S3 Fig — (A-C). Confocal images of wild type (A) and mbl-1(0) (B) worms expressing a presynaptic reporter Pmec-7-ELKS-1::TagRFP (jsIs1075), shown in magenta color. The neuron is also labeled with diffusible reporter Pmec-7-GFP (muIs32), shown in green. The ectopic synapse in the PLM anterior process in the mbl-1(0) background is marked by a red arrowhead, whereas the original synapse is marked by a white arrowhead. (C) The histogram shows the percentage of PLM neurons with ectopic synapses in the mbl-1(0) background. N = 3 independent replicates, n (number of worms) = 25–30. (D-F) Representative images (D) and schematic (E) of D-type motor neurons labeled with Punc-25::GFP (juIs76) in the wild-type and mbl-1(0) at the L4 stage. Red arrow showing a defect in neurite growth in mbl-1(0) background. (F) The histogram shows the percentage of worms showing neurite defects in the mbl-1(0). N = 3–4 independent replicates, n (number of worms) = 30–50. (G) Representative confocal images of touch neurons in the mutants of different RNA binding proteins. The names of the mutants are mentioned next to the respective image panels. (H) Quantification of any defects in PLM morphology seen in the mutants mentioned in (G) N = 3 independent replicates, n (number of worms) = 80–130. For C, F, and H ***P <0.001; Fisher’s exact test. ns, not significant. (TIF) [file pgen.1010885.s003.tif]

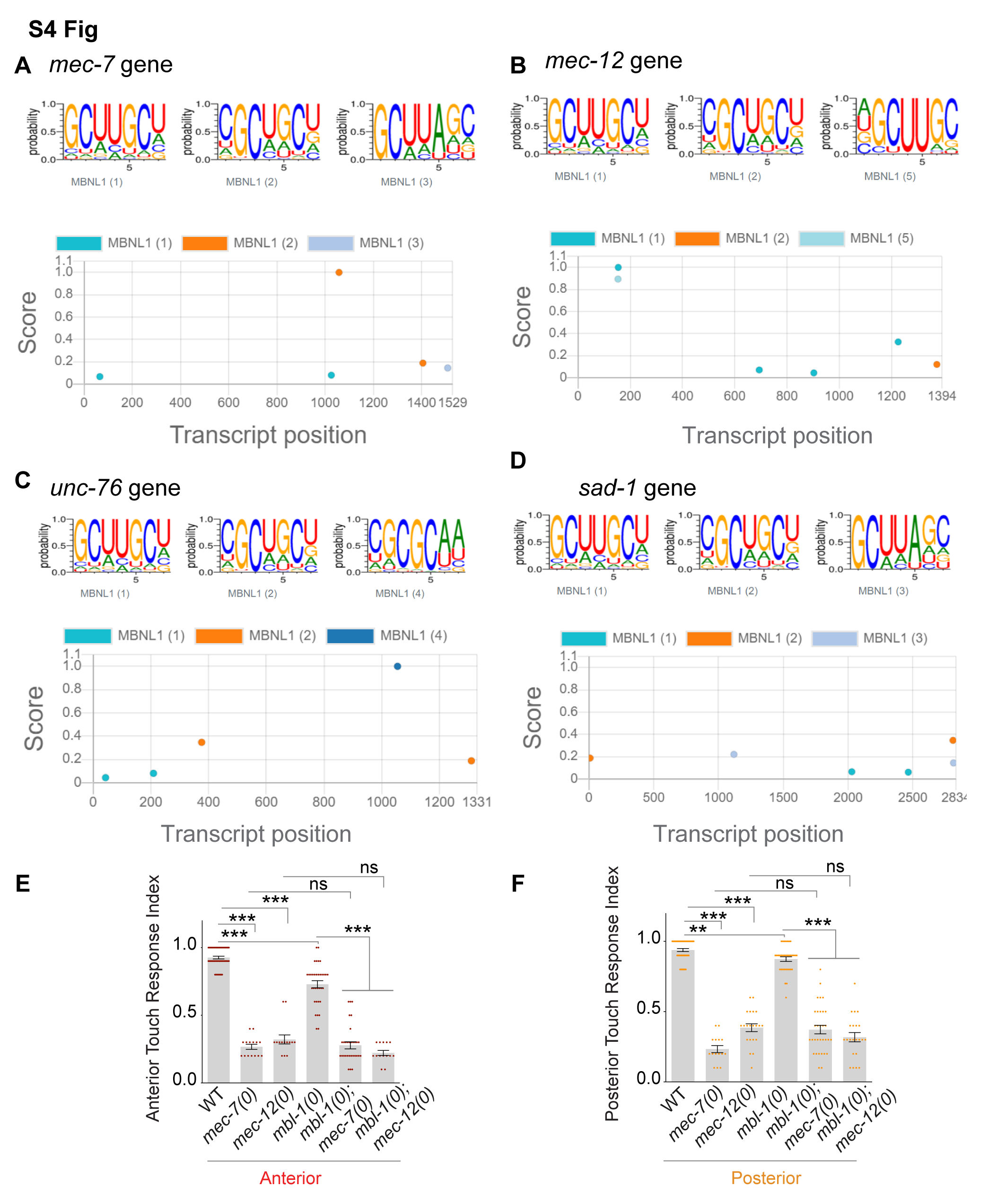

Supplement: S4 Fig — (A-D) Pictures depicting MBL-1/MBNL-1 preferential binding sequence and binding positions in the transcript of mec-7 (A), mec-12 (B), unc-76 (C), and sad-1 (D). (E and F) The histograms show the anterior (E) and posterior (F) gentle touch response index of the worm in the wild-type, mbl-1(0), mec-7(0), mec-12(0), mbl-1(0) mec-7(0) and mbl-1(0); mec-12(0) backgrounds. N = 3 independent replicates, n (number of worms) = 16–50. For E-F, **P < 0.01; ***P < 0.001. Error bars represent SEM. Statistical comparisons were done using ANOVA with Tukey’s multiple comparison test. ns, not significant. (TIF) [file pgen.1010885.s004.tif]

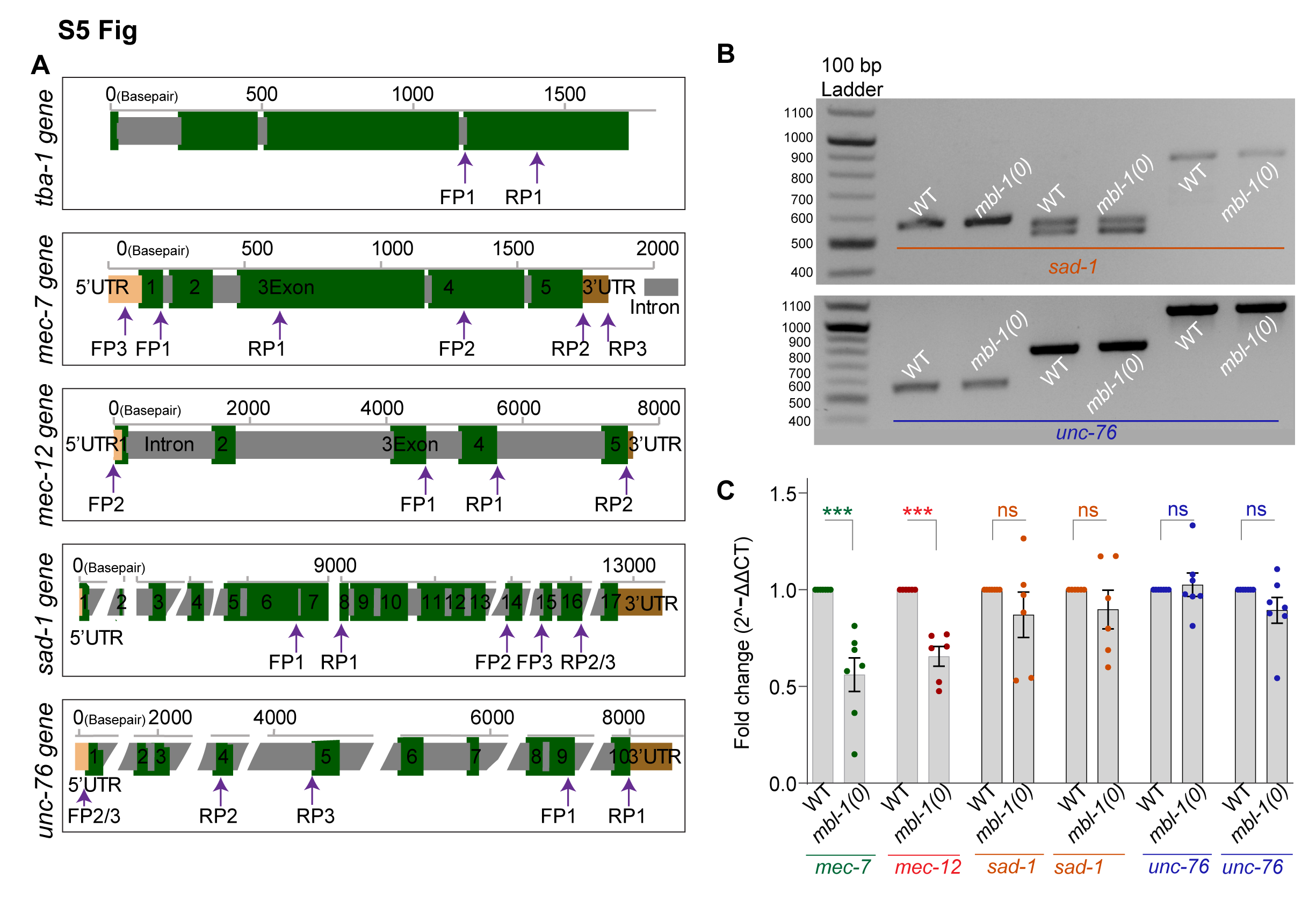

Supplement: S5 Fig — (A) Illustration showing the positions of different primers, used for checking the transcript length or doing qRT-PCR, on tba-1, mec-7, mec-12, sad-1, and unc-76 genes. The sequence of these primers is given in the supplementary file S4 Table. (B) Representative agarose gel image showing sad-1 and unc-76 transcript in the wild-type and the mbl-1(0) background. (C) The histogram is showing the relative fold change of the transcript of mec-7, mec-12, sad-1, and unc-76 in the wild type and the mbl-1(0) backgrounds. These data were obtained from quantitative real-time PCR (qRT-PCR). Independent replicates (N) = 6 and the number of reaction (n) = 6–8. For C, ***P < 0.001; ANOVA with Tukey’s multiple comparison test. Error bars represent SEM. ns, not significant. (TIF) [file pgen.1010885.s005.tif]

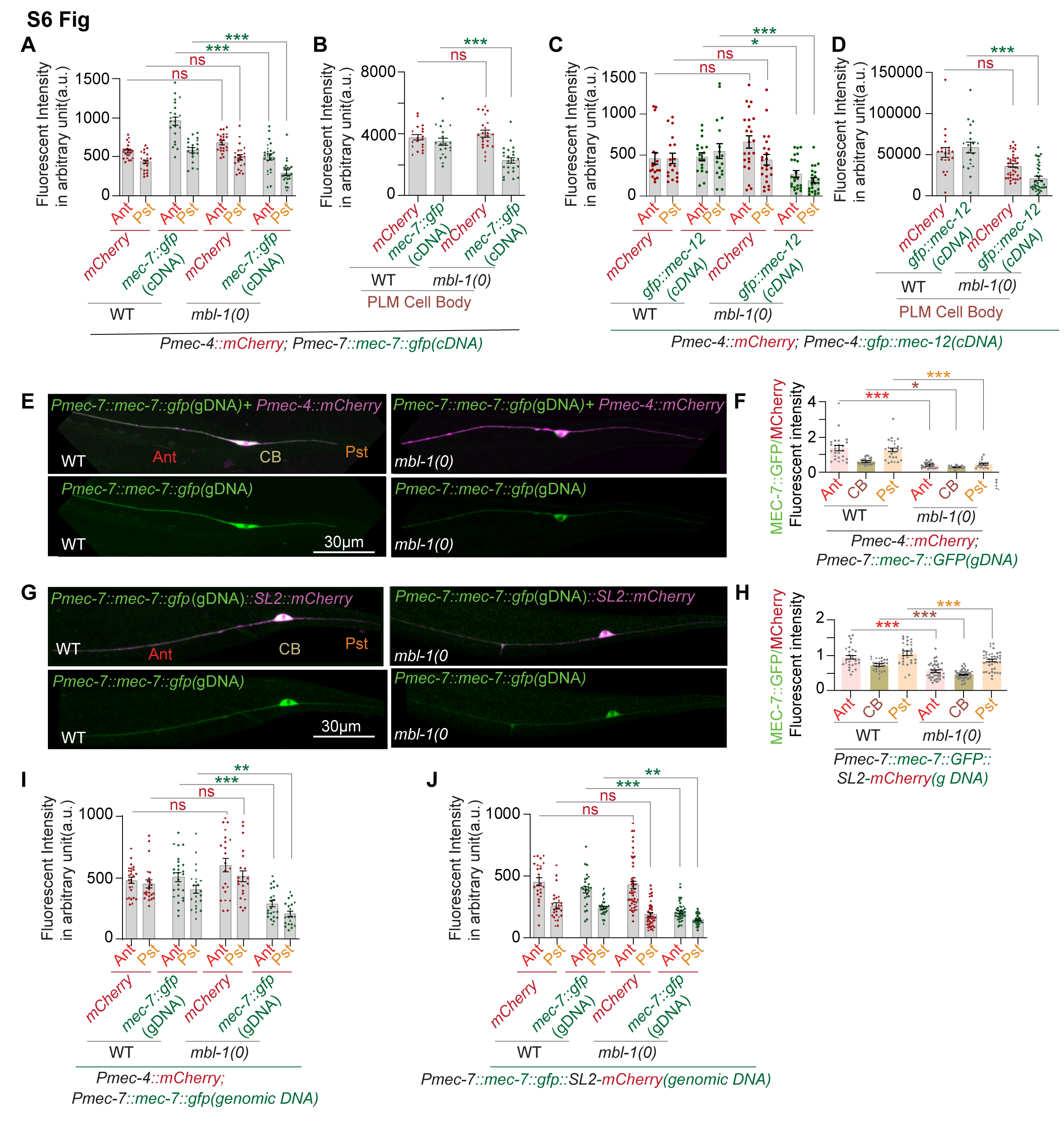

Supplement: S6 Fig — (A-D) Histograms showing the absolute fluorescence intensities of Pmec-7::mec-7::gfp (cDNA) (shrEx473) (A-B), Pmec-4::gfp::mec-12 (cDNA) (shrEx492) (C-D), and Pmec-4::mCherry (tbIs222) (A-D) in the wild-type and mbl-1(0) backgrounds. The fluorescence intensity is quantified in an arbitrary unit from the anterior (Ant) and posterior (Pst) neurites of PLM, from 50 μm regions of interest (ROI) as shown in the Fig 6F, and the PLM cell body in the wild-type and mbl-1(0) backgrounds. For A-D, independent replicates (N) = 3–4 and the number of neurons (n) = 20–25. (E and G) Representative confocal images of the worms expressing Pmec-7::mec-7::gfp (genomic DNA) (shrEx474) and Pmec-4::mCherry (tbIs222) (E), and worms expressing Pmec-7::mec-7::gfp::SL2::mCherry (shrEx486) (G) in wild-type and mbl-1(0) background. (F and H) The histogram is showing quantification of the ratio (MEC-7::GFP/MCherry) of average fluorescent intensity from 50 μm regions of interest (ROI) as shown in Fig 6F in the anterior (Ant) and posterior (Pst) neurites and cell body (CB) of PLM neurons. (I-J) Histograms showing the fluorescence intensities of Pmec-7::mec-7::gfp (genomic DNA) (shrEx474) and Pmec-4::mCherry (tbIs222) in arbitrary units (I) and histogram showing fluorescence intensities of Pmec-7::mec-7::gfp::SL2::mCherry (shrEx486) (J) in the wild-type and mbl-1(0) backgrounds. For F, H, I, and J, independent replicates (N) = 3–4 and the number of neurons (n) = 20–25. For A-D, F, H, and I-J, *P < 0.05; **P < 0.01; ***P <0.001; ANOVA with Tukey’s multiple comparison test. Error bars represent SEM. ns, not significant. (TIF) [file pgen.1010885.s006.tif]

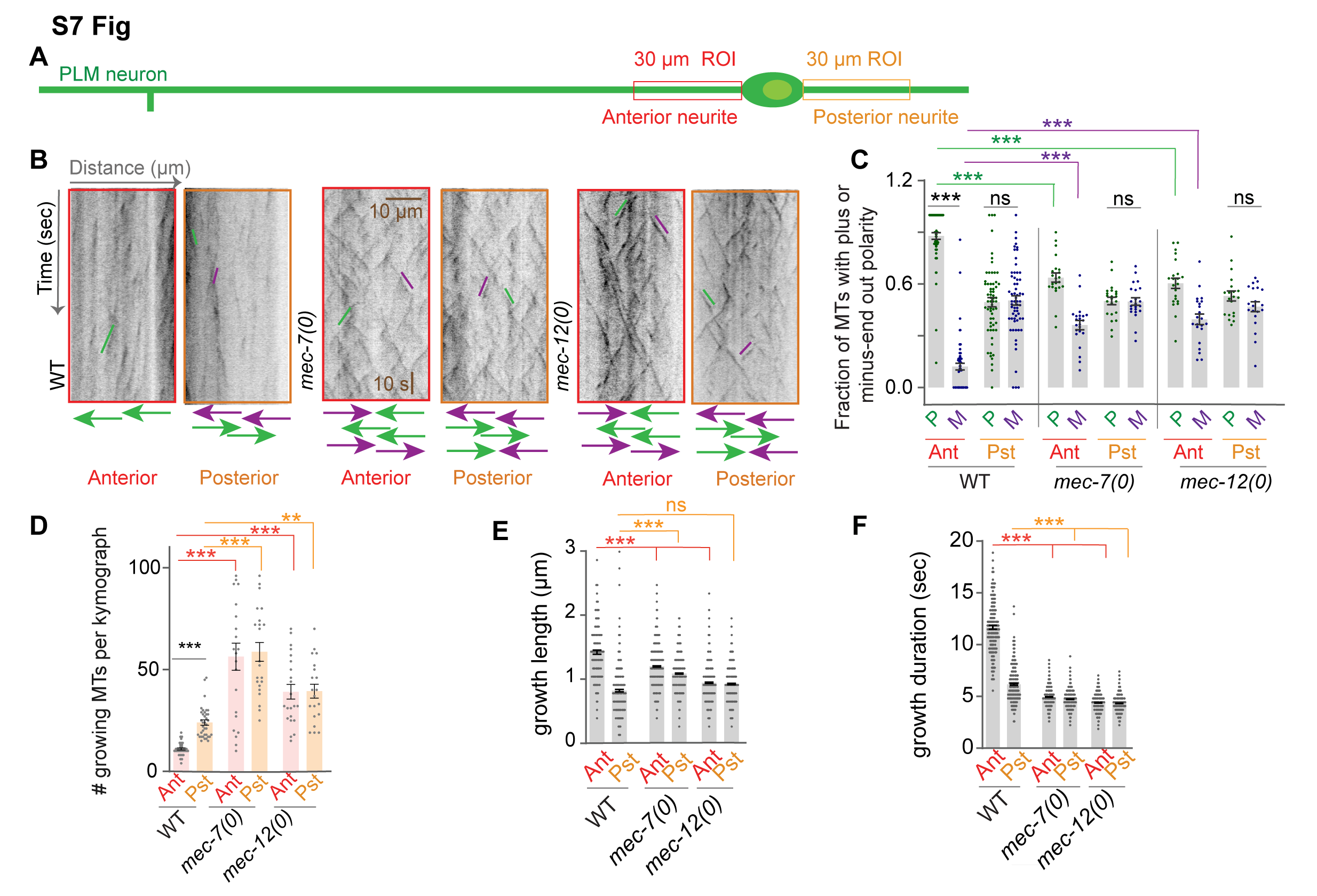

Supplement: S7 Fig — (A) Schematic of the PLM neuron showing the 30 μm Regions of interest (ROIs), marked in red and orange for anterior and posterior neurites, respectively. These ROIs were used for analyzing the time-lapse movies of Pmec-4::EBP-2::GFP (juIs338) in wild-type, mec-7(0), and mec-12(0) backgrounds. (B) Representative kymographs of EBP-2::GFP obtained in the wild-type, mec-7(0), and mec-12(0) backgrounds obtained from the above-mentioned ROIs. The green and magenta traces on kymographs represent the Plus-end-out (microtubule growth events away from the cell body) and Minus-end-out (towards the cell body) tracks, respectively. (C) The bar graph is showing the fraction of microtubules with plus-end-out’ (P) or ‘minus-end-out’ (M) polarity in wild-type, mec-7(0), and mec-12(0) backgrounds in the PLM anterior (Ant) and posterior (Pst) neurites. N = 3–5 independent replicates, n (number of worms) = 20–50. (D) The bar graph represents the number of EBP-2::GFP tracks (number of growing microtubules) in PLM anterior (Ant) and posterior (Pst) neurites in wild-type, mec-7(0), and mec-12(0). N = 3–5 independent replicates, n (number of worms) = 20–50. (E and F) Growth length (E) and growth duration (F) of the tracks, measured from net pixel shift in the X and Y axis, respectively, from kymographs shown in B. N = 3–5 independent replicates, n (number of tracks) = 1986–6064. For C-F, ***P < 0.001; ANOVA with Tukey’s multiple comparison test. Error bars represent SEM. ns, not significant. (TIF) [file pgen.1010885.s007.tif]
