# Supplementary material for: Muscleblind-1 interacts with tubulin mRNAs to regulate the microtubule cytoskeleton in C. elegans mechanosensory neurons
Source: PLoS Genet. 2023 Aug 21;19(8):e1010885. doi: 10.1371/journal.pgen.1010885 (PMC10470942; doi:10.1371/journal.pgen.1010885)
Supplement: Attachment — Submitted filename: Response Letter-Puri etal-Revision-210523.pdf [file pgen.1010885.s014.pdf]

Anindya Ghosh Roy, PhD
Associate Professor, Wellcome Trust-DBT Intermediate Fellow

National Brain Research Center (Deemed University)
(An Autonomous Institute of Deptt. of Biotechnology, Ministry of Science and Technology, Govt. of India)
NH-8, Nainwal Mode, Manesar-122051 (Haryana)

Point wise response

Dated, 21st May 2023, Gurgaon

Re: "Muscleblind-1 regulates microtubule cytoskeleton in C. elegans mechanosensory neuron through tubulin mRNAs" (PGENETICS-D-22-01039) by Puri et al.

We thank the editors to consider our manuscript further for the review process. We thank the reviewers for their valuable inputs for the betterment of the manuscript. We have addressed all the points clearly and new experimental results were provided wherever suggested.

Editor's note:

Dear Dr. Ghosh-Roy,

Thank you very much for submitting your Research Article entitled 'Musleblind-1 regulates microtubule cytoskeleton in *C. elegans* mechanosensory neuron through tubulin mRNAs' to PLOS Genetics.

The manuscript was fully evaluated at the editorial level and by three independent peer reviewers. The reviewers appreciated the attention to an important problem, but raised some substantial concerns about the current manuscript that need to be addressed to provide mechanistic insights, expand the data analysis, and strengthen the conclusions. These are especially related, but not limited, to the insufficient data provided to support the conclusion that MBL-1 stabilizes the *mec-7* and *mec-12* transcripts, how *mbl-1* suppresses *kpl-7*, and what link exists between the regulation of *mec-7/12* by *mbl-1* and the microtubule polarity and dynamics.

Response: Thanks for the positive comments on the manuscript. We have revised the experiments and analysis substantially to elucidate the mechanism of how MBL-1 regulates the transcripts of tubulin mRNAs by binding to these. These data strongly indicated the role of MBL-1 in the optimization of the expression of *mec-7* and *mec-12* mRNAs, see the data in Fig 6, and 7, and the discussion section (page 23, lines 536-549).

We have elaborated the explanation and the result section explaining why microtubule dynamics and polarity is affected in *mbl-1* mutant, due to the reduced level of tubulin subunits in touch neuron see the data in Fig 3 and Fig S7B-F and the discussion section (pages 24, 560-563).

Telephone: +91-124-2845 237 Fax : +91-124-2338910
Email : anindya@nbrc.ac.in **Website : www.nbrc.ac.in**

Anindya Ghosh Roy, PhD
Associate Professor, Wellcome Trust-DBT Intermediate Fellow

National Brain Research Center (Deemed University)
(An Autonomous Institute of Deptt. of Biotechnology, Ministry of Science and Technology, Govt. of India)
NH-8, Nainwal Mode, Manesar-122051 (Haryana)

Moreover, the manuscript will need to be structured within the context of previous published work, some of which has not been cited. This includes the early work on neuronal development and polarization, as indicated by Reviewer 1, as well as the work on *mbl-1* in synaptic formation(Shen lab) that should be presented as the entry point for this study of *mbl-1* on synapses.

Response: We have added the suggested references in the introduction and other appropriate sections.

Finally, the manuscript will need to be carefully checked for grammatical errors, typos, and colloquial language, which populate the current version. Based on these reviews, we will not be able to accept this version of the manuscript, but we would be willing to review a much-revised version. We cannot, of course, promise publication at that time.

Response: We have revised the manuscript thoroughly for language and clarity.

Should you decide to revise the manuscript for further consideration here, your revisions should address the specific points made by each reviewer. We will also require a detailed list of your responses to the review comments and a description of the changes you have made in the manuscript.

Reviewer's Responses to Questions

Comments to the Authors:
Please note here if the review is uploaded as an attachment.

Reviewer #1: The article by Puri et al. investigates the role of MBL-1 in neuronal structure and synapse localization in the mechanosensory neurons of C. elegans. It provides new insights into the importance of this molecule for correct neuronal structure. It also offers mechanistic insights by demonstrating interactions with mec-7, mec-12, and sad-1 mRNAS. Thus, overall it provides novel data on how an RNA binding protein interacts with cytoskeletal elements to mediate correct neuronal structure. As listed below, I have numerous suggestions for the authors to address before the manuscript can be considered for publication.

Overall, the writing of the manuscript needs significant improvement. The grammar and sentence structure need to be improved to help with the readability of the manuscript, as there are many instances in which the meaning of the authors is unclear.

It would be important to cite some of the early work on neuronal development and polarization in C. elegans in the introduction. For example, Adler et al., 2006 Nat Neuro; Chang et al., 2006 Curr Biol; Hilliard and Bargmann, 2006 Dev Cell; Prasad and Clark, 2006 Development; Quinn et al., 2008 Curr Biol.

Anindya Ghosh Roy, PhD
Associate Professor, Wellcome Trust-DBT Intermediate Fellow

National Brain Research Center (Deemed University)
(An Autonomous Institute of Deptt. of Biotechnology, Ministry of Science and Technology, Govt. of India)
NH-8, Nainwal Mode, Manesar-122051 (Haryana)

All gene names should be italicized.

Response- We would like to thank the reviewer for his constructive comments on our manuscript. We have incorporated the above-suggested citations (line 98-101, page 5). Please see the response for each comment below.

Figure 1.
Panel A. The authors may wish to label the PVM neurons in their images to make them more understandable for those who are not familiar with the mec neurons of C. elegans.

Response- Thanks for the suggestion, we have labeled it in the revised manuscript. Please see Figure 1 A.

Panel C. Was the ALM defect analysed with a yes or no qualification, ie it either had a posterior neurite or it didn't? This is not clear in the manuscript.

Response- Thanks for your query and we are sorry that we did not make it clear in the text. Yes, the ALM defect was analyzed as if it either had a posterior neurite defect or it did not. The ALM posterior neurite when it reaches or crosses vulva was quantified as "ectopic growth" (Figure 1A & C). We have clearly mentioned it in the methods section "Widefield fluorescence imaging of mechanosensory neurons for quantifying developmental defects". Please see lines 647-658, page 27-28.

Panel D and E. The stats are not clear in these two graphs – is the fosmid rescue line significantly different to the controls (ie WT and mbl-1(ju1128)?

Response- We have presented the statistics in 1D-E panels with more clarity. In the fosmid rescue line, the normalized length of the PLM posterior neurite is significantly different from the controls. However, there is no significant difference in the case of the PLM anterior neurite. Please see the revised statistics in Figure 1 legend. Please note that in the revised manuscript, we referred to the 'fosmid' as 'genomic *mbl-1*' as it exclusively contains the *mbl-1* locus.

Is the fosmid able to rescue the tm1563 allele?

Response: Please see the revised Figure 1C-E. The fosmid is able to rescue the suppression phenotype in *klp-7(tm2143); mbl-1(tm1563)* background. This is similar to what we observed in *klp-7(tm2143); mbl-1(ju1128)* background. Please also see in Figure-2 that fosmid (genomic *mbl-1*) is able to rescue all the phenotypes in *mbl-1* mutant irrespective of the presence of *klp-7* mutant in the background.

Figure 2.

Telephone: +91-124-2845 237 Fax : +91-124-2338910
Email : anindya@nbrc.ac.in **Website : www.nbrc.ac.in**

Anindya Ghosh Roy, PhD
Associate Professor, Wellcome Trust-DBT Intermediate Fellow

National Brain Research Center (Deemed University)
(An Autonomous Institute of Deptt. of Biotechnology, Ministry of Science and Technology, Govt. of India)
NH-8, Nainwal Mode, Manesar-122051 (Haryana)

Why did the authors choose to focus on the tm1563 allele for this figure (for images and rescue) when they primarily used the ju1128 allele in Figure 1?

Response- We have characterized both the alleles for the touch neuron phenotypes. We found that both of these alleles give the same penetrance for the TRN phenotypes (Figure 2B). We have also shown in the revised Figure1 that the genomic copy of *mbl-1* rescues the suppression of *klp-7* mutant phenotype in *klp-7(tm2143); mbl-1(tm1563)* background. Then for the rest of the figures, we focused on using the *tm1563* allele. There was no other specific reason for using the *tm1563* allele.

Panel A. In the image of the mbl-1 mutant, the ALM and PLM axons appear much fainter than the WT and possibly fragmented. Is there degeneration occurring in these neurons? Thus, is the short phenotype developmental or is it degenerative?

Response- We carefully looked for degeneration phenotype in *mbl-1* mutant. We did not find any neurite fragmentation till day-6 adulthood. We see that about 33% PLM neurites in *mbl-1* mutant show neurite fragmentation at the day-7 adult stage and the same amount of degeneration is also seen in wild-type background at this age (See Figure S2G-I). We also revised Figure-2 to give the best possible description of the mutant phenotypes. We have replaced the old image with a better clear image (Figure 2A). Please also see the relevant description in line-193-197 in page 9.

Panel B. It's interesting that the TRN-specific rescue is not as strong as the genomic or pan-neuronal rescues. Why do the authors think this is so? Is this difference significantly different?

Response- Thanks for the query. We have compared the rescue between genomic or pan-neuronal *mbl-1* constructs with the touch neuron-specific *mbl-1.* There is no significant difference in the phenotype among the different rescue strains (Figure 2B).

Panel F. The text states that "MBL-1::GFP was 238 highly enriched in many neurons including ALM and PLM touch neurons". However, from the images provided it is impossible to see the PLM neurons. It appears that the authors have performed the required co-localization experiments using a mCherry marker, but this is not visible in these images. Please provide clearer and/or enlarged images and/or quantification to demonstrate that MBL-1 is expressed in the ALM and PLM neurons.

Response- We have revised the F panel in Figure 2. We have also provided the inset images of the PLM and ALM neuron clearly showing the presence of MBL-1::GFP in the neurites (arrowheads, Figure 2F).

Furthermore, do you find enrichment of MBL-1 in any areas of these neurons?

Response- We did not find any area/location-specific enrichment of MBL-1 in TRNs. It is present in soma as well as in the anterior and posterior neurites of PLM and ALM neurons Figure 2F.

Telephone: +91-124-2845 237 Fax : +91-124-2338910
Email : anindya@nbrc.ac.in **Website : www.nbrc.ac.in**

Anindya Ghosh Roy, PhD
Associate Professor, Wellcome Trust-DBT Intermediate Fellow

National Brain Research Centre NBRC

National Brain Research Center (Deemed University)
(An Autonomous Institute of Deptt. of Biotechnology, Ministry of Science and Technology, Govt. of India)
NH-8, Nainwal Mode, Manesar-122051 (Haryana)

Figure 3.
The author may wish to consider separating this figure into two to make it easier to follow.

Response- Thanks for the suggestion. We have separated this figure into Figure 3 (describing the microtubule-related data) and Figure 4 (describing the axonal transport-related data). Please see the revised Figure-3 and Figure-4.

Panel M/N. The functional decline in the posterior response is quite subtle. It would be good to show that this is rescued with the introduction of the rescue transgene to provide further strength to the findings. Expanding subsequent findings, especially with mec-12, mec-7, and sad-7, to assess their effects on the mbl-1-mediated reductions in touch response would provide further strength to the proposed interactions and their mechanisms of action.

Response- Thanks for the suggestions. We have performed a touch assay in the rescue background which has *mbl-1* transgene. The transgene could rescue the defects of both anterior and posterior touch sensation in *mbl-1(tm1563)* background (Figure 4G-H).

We have also done touch assay for *mbl-1(tm1563) mec-7(0)* and *mbl-1(tm1563); mec-12(0).* The *mbl-1(tm1563) mec-7(0),* and *mbl-1(tm1563); mec-12(0)* double mutants have similar gentle touch sensation defect as single mutants of *mec-7* and *mec-12* respectively (Figure S4E-F). This data suggests that *mbl-1* might be regulating gentle touch sensation through *mec-7* and *mec-12*.

Figure 4.
Both mec-7 and mec-12 mutants have previously been shown to present a high penetrance of axonal degeneration in the mec neurons. However, here the authors again refer to the phenotype as short neurites. The authors need to include additional experiments that clarify their phenotype as either developmental or degenerative.

Response: As discussed in the previous comments, we did not see the degeneration phenotype in the *mbl-1* mutant until day 6. The short neurite phenotype seen in L4 and earlier stages in the *mbl-1* mutant is not related to degeneration. Similarly, there are already reports that suggest that *mec-7* and *mec-12* mutants have outgrowth defects (Lee et al MBoC 2021, Zheng et al MBoC 2017, Lockhead et al MBoC 2016). In this report, we tried addressing the roles of MBL-1 based on the effects of its loss of function mutation on neurite outgrowth, synapse defects, microtubule dynamics, etc. There could be a degeneration aspect in older stages, which is not the focus here. We have put the relevant references for the roles of *mec-7/12* in neurite outgrowth in the introduction section (line-98-101, page-4) and the discussion section as well (line-563-565, page-24).

Telephone: +91-124-2845 237 Fax : +91-124-2338910
Email : anindya@nbrc.ac.in **Website : www.nbrc.ac.in**

Anindya Ghosh Roy, PhD
Associate Professor, Wellcome Trust-DBT Intermediate Fellow

National Brain Research Center (Deemed University)
(An Autonomous Institute of Deptt. of Biotechnology, Ministry of Science and Technology, Govt. of India)
NH-8, Nainwal Mode, Manesar-122051 (Haryana)

Figure 5.
Panel D-E. I'm not sure that I follow the authors assumption that "This reduction observed in the mbl-1(0) mutants as compared to the wild type is interesting, as it illustrates the fate of the pre-existing transcripts in the absence of MBL-1 and in the absence of any new transcription. As transcription was blocked, the observed additional decrease could be attributed to the increased instability of the pre-existing transcripts in the absence of MBL-1." It seems that the more plausible explanation would be that this experiment is showing the normal turn-over of the transcripts, as for both WT and mutant you show an ~40% decrease after treatment. Thus, the mutant is appearing to behave the same as the WT after treatment. As such, I am not convinced by the conclusion that "MBL-1 is regulating the stability of mec-7 and mec-12 mRNA in PLM neurons."

Response- We have made it clear now, please see the revised Figure 6 and explanation page 17-18, line 387-412 .

We have compared *mec-7* and *mec-12* mRNA levels in the presence or absence of Act D in both WT and *mbl-1(0)* backgrounds. *mec7/ mec-12* transcript levels in the presence of Act D were normalized to *mec-7/mec-12* levels in the absence of Act D in both WT and *mbl-1(0)* mutants.

In the WT background, there was a significant reduction in *mec-7/mec-12* transcript levels in the presence of Act D. However the *mbl-1(0)* mutants showed further reduction of *mec-7/mec-12* transcript levels upon Act D treatment. It is important to note that, Act D-dependent reduction of *mec-7/mec-12* transcript levels in the wild-type background indicates their inherent turnover. If *mbl-1* was not influencing *mec-7/mec-12* transcript stability, the expected degradation pattern should have been similar in both WT and *mbl-1(0)* mutant backgrounds. However, this is not the case.

The fold reduction in *mec-7* transcript due to ActD treatment in wild-type vs *mbl-1* mutant is 0.7462 (25 %) vs 0.4170 (58%). Similarly, the fold reduction in *mec-12* transcript in wild-type vs *mbl-1* mutant is 0.8245 (18%) vs 0.6689 (33%). And these are all statistically significant (see figure-6D-6E, and it's legend, also shown below is 6E) Therefore, we believe that the enhanced degradation of *mec-7/mec-12* transcripts detected in the *mbl-1(0)* mutant upon Act D treatment is indicative of MBL-1 influencing the stability of these transcripts. Please also see the revised text for a better explanation (line-387-412, Page16-17)

Telephone: +91-124-2845 237 Fax : +91-124-2338910
Email : anindya@nbrc.ac.in **Website : www.nbrc.ac.in**

Anindya Ghosh Roy, PhD
Associate Professor, Wellcome Trust-DBT Intermediate Fellow

National Brain Research Center (Deemed University)
(An Autonomous Institute of Deptt. of Biotechnology, Ministry of Science and Technology, Govt. of India)
NH-8, Nainwal Mode, Manesar-122051 (Haryana)

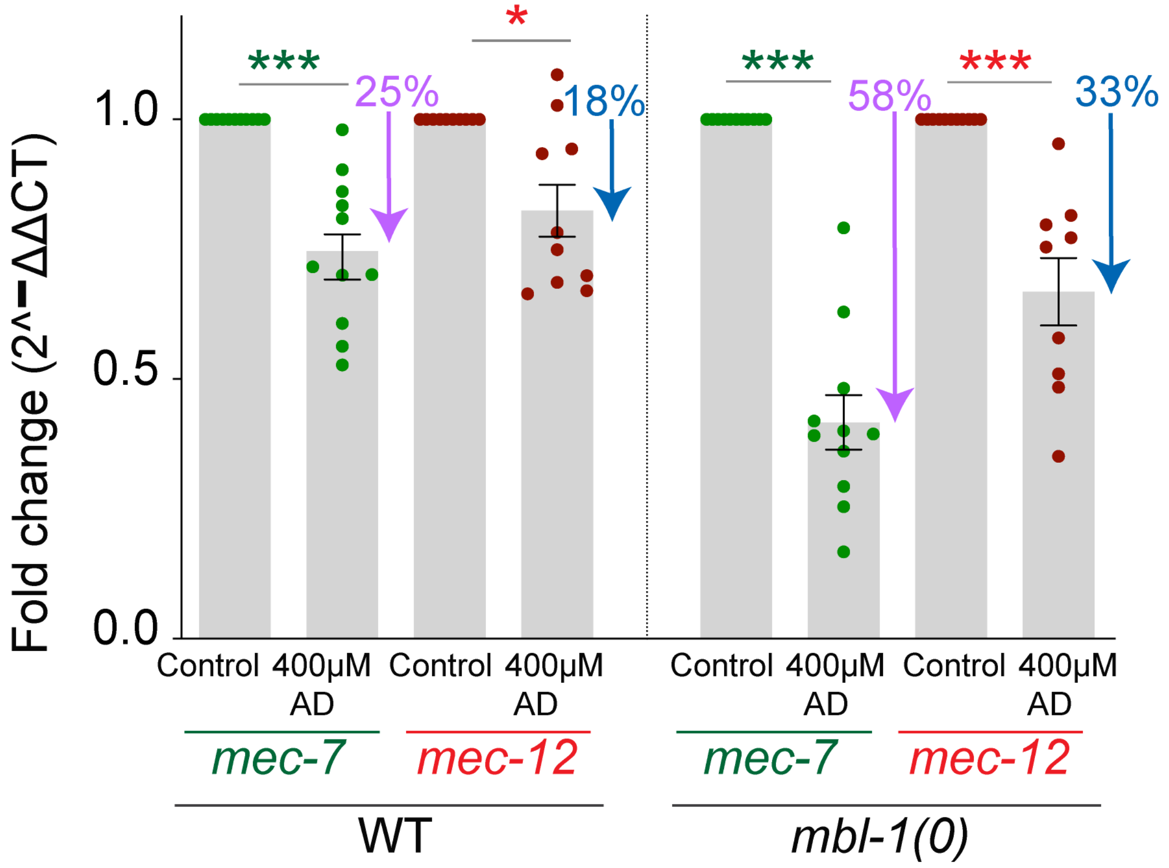

Panel F/G. Did the authors find similar results with mec-12?

Response- Please see the revised Figure 6G and I and also Figure S6B, 6C, we have added the data from the reporter P*mec-4*-GFP::MEC-12. This shows a similar trend in the reduction of GFP::MEC-12 intensity in *mbl-1* mutant. Please also see the revised text for a better explanation (line-423-425, Page-18)

Figure 6.
Panel B/C. These panels show interaction with mec-7, mec-12 and sad-1, but then mec-12 appears to be forgotten about for the next experiments in this figure and in the text, which only refers to mec-7 and sad-1. Why is this? Did the authors find that mec-12 did not interact with MBL-1 in the mec neurons in subsequent experiments? The experiments conducted on mec-7 and sad-1 in panels D-E should also be included for mec-12.

Anindya Ghosh Roy, PhD
Associate Professor, Wellcome Trust-DBT Intermediate Fellow

National Brain Research Center (Deemed University)
(An Autonomous Institute of Deptt. of Biotechnology, Ministry of Science and Technology, Govt. of India)
NH-8, Nainwal Mode, Manesar-122051 (Haryana)

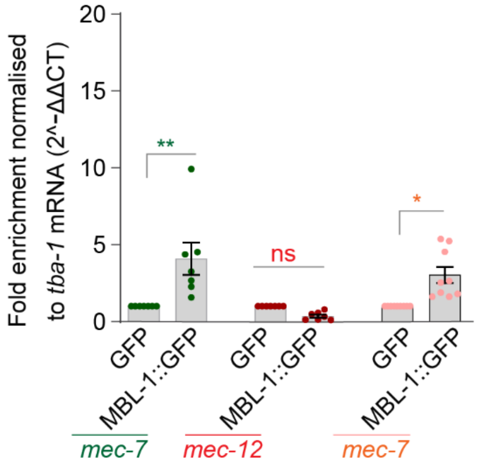

Response- We have done all of these experiments. Please see the results above. Although we successfully pulled down *mec-12* mRNA along with the *Pmbl-1*:FLAG::MBL-1::GFP, *mec-12* mRNA was not significantly enriched when we immunoprecipitated MBL-1::GFP(C isoform cDNA) from the transgenic strain expressing *Pmec-4*::MBL-1(C-isoform)::GFP. A genomic copy of *mbl-1* expresses all possible isoforms, which most likely includes the isoform binding to *mec-12* mRNA. However, for MBL-1 pull down from mechanosensory neurons we used a single isoform (also our cDNA rescue construct) available to us (obtained from Yuji Kohara collection, Japan). No other isoform was available from Kohara collection and our effort to clone the other isoforms failed as of now. Therefore, we could not try this immunoprecipitation experiment with any other isoform yet. However, specific enrichment of *mec-12* in the immunoprecipitation with FLAG::MBL-1::GFP tells mec-12 binds to MBL-1. Moreover, mec-12 is only expressed in touch neuron.

Telephone: +91-124-2845 237 Fax : +91-124-2338910
Email : anindya@nbrc.ac.in **Website : www.nbrc.ac.in**

Anindya Ghosh Roy, PhD
Associate Professor, Wellcome Trust-DBT Intermediate Fellow

National Brain Research Center (Deemed University)
(An Autonomous Institute of Deptt. of Biotechnology, Ministry of Science and Technology, Govt. of India)
NH-8, Nainwal Mode, Manesar-122051 (Haryana)

MBL-1 Isoforms

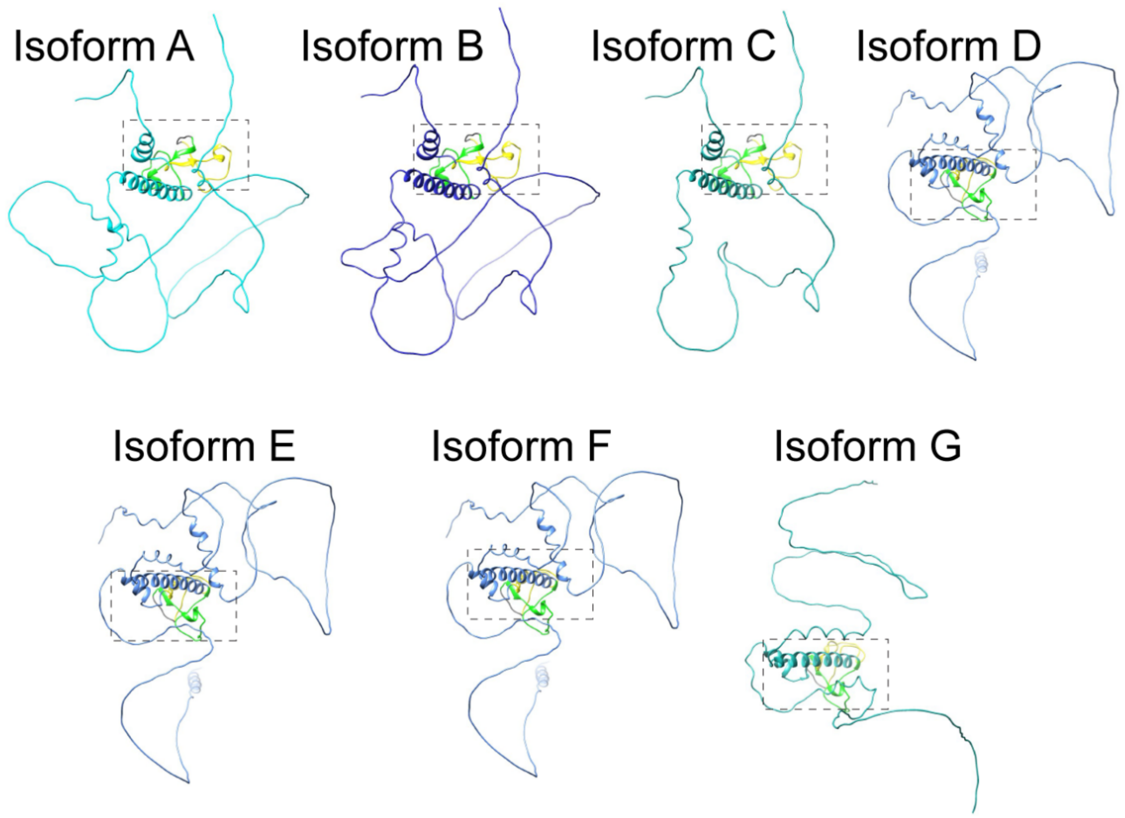

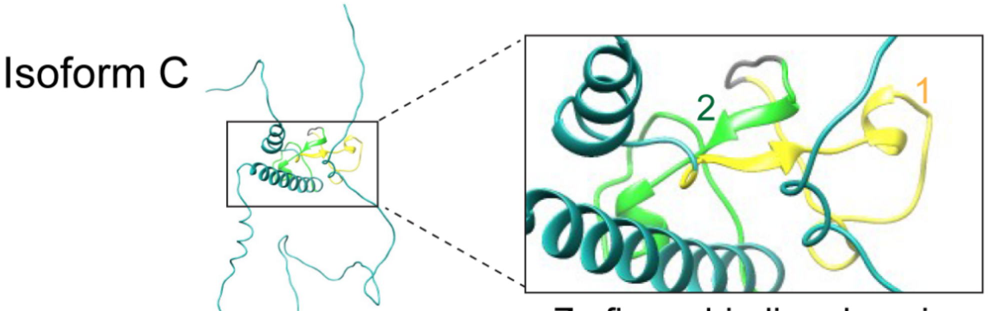

Zn finger binding domains

RBP Motifs(as motif logo) scanned by oRNAment database

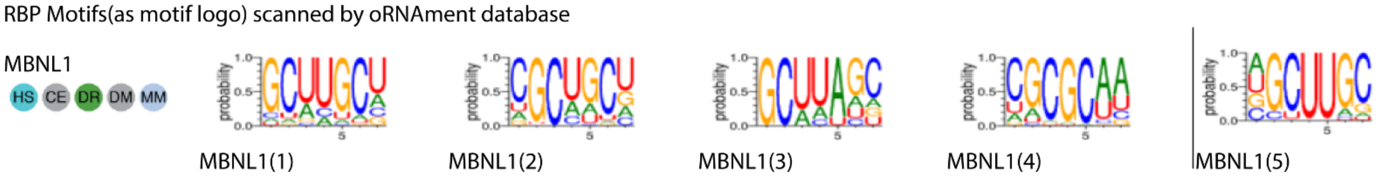

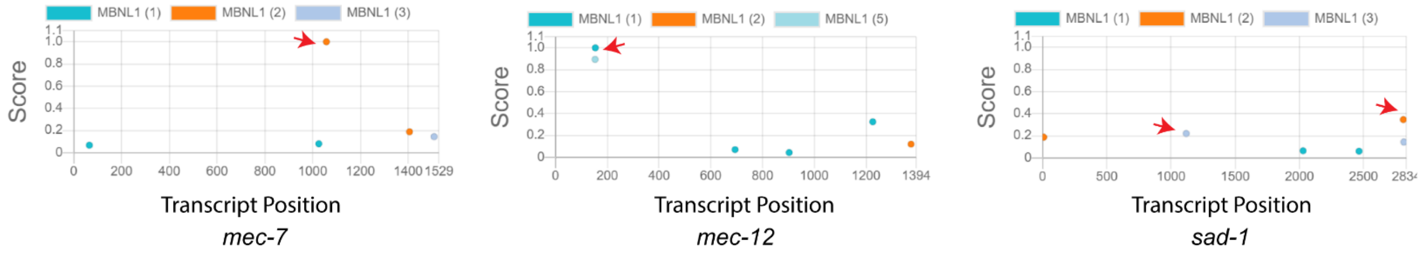

Anindya Ghosh Roy, PhD
Associate Professor, Wellcome Trust-DBT Intermediate Fellow

National Brain Research Centre
NBRC

National Brain Research Center (Deemed University)
(An Autonomous Institute of Deptt. of Biotechnology, Ministry of Science and Technology, Govt. of India)
NH-8, Nainwal Mode, Manesar-122051 (Haryana)

MBL-1 has A-G isoforms as annotated in wormbase. Though the RNA binding Zn finger domain aa sequence is conserved across these isoforms, the structural variations across these isoforms can have different binding preferences.

The putative binding motifs obtained from the *in-silico* analysis show that MBNL-1/MBL-1 may recognize five consensus sequences and the binding probabilities for each may vary in different transcripts (marked by the red arrow in the figure above). This might be the reason we are not pulling *mec-12* along with MBL-1::GFP (C isoform) from mechanosensory neurons.

Speaking concisely, *mec-12* transcript was not enriched in MBL-1::GFP (C isoform) pull down. This might be because of the low or no binding affinity of the MBL-1 C isoform for the MBNL-1 consensus sequence present in *mec-12.* See the (line-439-458, Page-18-19) for details. We also acknowledged and explained this in the discussion section (line-555-560, Page-24)

However, this does not alter the overall message of the manuscript that MBL-1 regulates microtubule cytoskeleton through the regulation of *mec-7* and *mec-12* mRNAs. We clearly established that MBL-1 limits the expression of *mec-7* and *mec-12* and therefore this impacts microtubule dynamics in touch neuron.

[References for isoform structure predictions:

- J
umper, J et al. Highly accurate protein structure prediction with AlphaFold. Nature (2021).)
- P
ettersen, E.F., Goddard, T.D., Huang, C.C., Couch, G.S., Greenblatt, D.M., Meng, E.C., and Ferrin, T.E. "UCSF Chimera - A Visualization System for Exploratory Research and Analysis." J. Comput.]

Figure 7.
Again, why wasn't mec-12 included in these analyses? It is included in the model in panel F. Although MEC-12 and MEC-7 are most commonly known as the major subunits of microtubules in the mec neurons, there are several examples in the literature demonstrating that they can function quite differently to one another and influence specific phenotype in different ways or to different degrees. Thus, it cannot be assumed that they will behave the same if the experiments have not been performed.

Response: Please see the revised Figure 8A-C panels. Expression of GFP::MEC-12 rescues the short axon phenotype in *mbl-1* mutant. Similarly in the wild-type background, GFP::MEC-12 expression in touch neuron causes overgrowth of the neurite as seen in the case of the overexpression of MEC-7::GFP. See the (line-462-476, Page-20) for details.

Panel F. The authors may wish to expand their model to make it more informative and reflective of their proposed mechanisms of action.

Anindya Ghosh Roy, PhD
Associate Professor, Wellcome Trust-DBT Intermediate Fellow

National Brain Research Center (Deemed University)
(An Autonomous Institute of Deptt. of Biotechnology, Ministry of Science and Technology, Govt. of India)
NH-8, Nainwal Mode, Manesar-122051 (Haryana)

Response- Thanks for the suggestion. We have made it more informative. Please see the revised Figure 8H.

Figure S2. The images are far too small to allow the reader to see the required detail. Yes, the schematics are of great assistance, but the original images need to be able to be seen.

Response- We have revised the image and separated this figure into Figure S2 and Figure S3. Please see these revised Figures.

The text states "In the mbl-1(0) mutant, we noticed that on the dorsal side, there are often gaps (red arrowhead, Figure S2J-L) indicating a synaptic defect". However, the authors have only used a diffuse GFP marker to visualise these neurons. Further analysis using a synaptic marker in these neurons is needed to conclude that these gaps indicate a synaptic defect rather than shorter neurites (similar to the phenotype in ALM/PLM).

Response- Thanks for the query. We have removed the words' synaptic defect. We have written it as a neurite growth defect as also suggested by the reviewer-2 in his/her minor comment #7. We also don't have any reagents to characterize the synaptic defect of motor neurons in our lab currently. Please see lines 212-217, Page 10.

Reviewer #2: Review_PGENETICS-D-22-01039

Puri, Ghosh-Roy et al. described how C. elegans MBL-1/Muscleblind, an RNA-binding protein, promotes axon growth and synapse development by regulating microtubule polarity through controlling the level of tubulin mRNAs. They first identified mbl-1 as a suppressor for touch neuron defects in the klp-7 Kinesin mutant, followed by providing evidence that microtubule dynamics and levels of touch neuron-specific tubulins (mec-7, mec-12) are altered in the mbl-1 mutant. RNA-IP experiments showed that MBL-1 binds mec-7 RNA, suggesting that MBL-1 sustains MEC-7 levels through a posttranscriptional mechanism. Consistent with this notion, overexpression of mec-7 rescued the neurite defects of the touch neurons in the mbl-1 mutant. Most of the experiments in this study were reasonable, although some clearly need further refinement in terms of data analysis. However, it remains unexplored how regulation of tubulins by MBL-1 is linked to microtubule polarity and dynamics in the touch neurons. The interaction between mbl-1 and sad-1 is not explored at a deeper level, making one wonder whether the molecular mechanisms by which mbl-1 regulates tubulins and sad-1 are similar or distinct. Such unaddressed issues compromise the mechanistic depth of the paper.

Response- We would like to thank the reviewer for his/her constructive comments. We have revised the manuscript with new experiments and revised data analysis. We have addressed in the discussion part how MBL-1 might be regulating tubulin to control microtubule polarity and

Telephone: +91-124-2845 237 Fax : +91-124-2338910
Email : anindya@nbrc.ac.in **Website : www.nbrc.ac.in**

Anindya Ghosh Roy, PhD
Associate Professor, Wellcome Trust-DBT Intermediate Fellow

National Brain Research Center (Deemed University)
(An Autonomous Institute of Deptt. of Biotechnology, Ministry of Science and Technology, Govt. of India)
NH-8, Nainwal Mode, Manesar-122051 (Haryana)

dynamics and also how MBL-1 is regulating *sad-1* kinase for the correct location of synapse positioning. Please see the response for each comment below.

In a previous article from our lab, we have shown that increased microtubule instability (dynamics) leads to the formation of mixed polarity microtubules. Whereas increased stability in microtubule supports uniparallel plus end-out organization of MTs in the anterior neurite of PLM (Puri et al 2021 JCB, reference # 7 in the current article).

In this report, we are showing that in *mbl-1* mutant microtubule instability is increased causing mixed polarity organization of microtubules. In our view, a reduced amount of *mec-7* and *mec-12* tubulin-subunits in *mbl-1* mutant leads to decreased polymerization rate (or increased depolymerization) leading to increased dynamics of microtubules, which in turn causes mixed polarity in microtubules. We added to evidence that independently *mec-7* and *mec-12* mutants show increased microtubule dynamics and mixed polarity organization of microtubules in the anterior neurites of PLM

Major points:

1. The paper needs a thorough editing check to correct the numerous grammatical errors and typos in writing. The authors should be more careful in checking their submitted manuscript. In addition, the authors have a tendency of using terms that are either too casual, or seem alienated to the audience without explanation. For example, in line 311 and line 314, “mode value” and “fraction polarity value” were used to describe the polarity defects of the mbl-1 mutant. I suggest the authors revise all terms like these to make their paper better received by the audience.

Response- Thanks for the suggestion. We have thoroughly revised the text for clarity and language. We have explained most of the terms which we have used and revised the text. As indicated here, in the description of phenotypes involving microtubule dynamics and polarity, we have simplified the data presentation and description. See the (line-233-268, Page 10-12) for details.

2. The suppression of klp-7 mutant phenotypes by mbl-1 remains unexplained. The authors need to test the following: (1) Whether microtubule dynamics in the klp-7 are different from the WT and the mbl-1 mutant, and whether defects of microtubule dynamics in the klp-7 mutant are suppressed by the mbl-1 mutation; (2) Whether mec-7 and mec-12 levels are altered by the klp-7 mutation, and whether these defects are again corrected by adding the mbl-1 mutation.

Response-We have previously shown that loss of *klp-7* leads to increased microtubule stability. This is reflected in the decreased number of EBP-2 tracks in the kymograph and the increased growth duration/length of the EBP-2 tracks (Puri et. al., 2021, JCB) (Also see the data in Figure 3). In contrast, the *mbl-1(0)* mutant shows more number of EBP-2 tracks with decreased growth duration/length (Figure 3D-F). Additionally, we found that the growth duration and length

Anindya Ghosh Roy, PhD
Associate Professor, Wellcome Trust-DBT Intermediate Fellow

National Brain Research Center (Deemed University)
(An Autonomous Institute of Deptt. of Biotechnology, Ministry of Science and Technology, Govt. of India)
NH-8, Nainwal Mode, Manesar-122051 (Haryana)

parameters are reduced in *klp-7(0); mbl-1(0)* double mutant as compared to the *klp-7(0)* single mutant which shows that the *mbl-1(0)* mutation partially suppresses the increased stability phenotype of the microtubules in the *klp-7(0)* background (Figure 3E-F). See the (line-233-268, Page 10-12) for details.

Similar to the *mbl-1* mutant, both *mec-7* and *mec-12* mutants have increased microtubule dynamics (Figure S7 B-F). This is consistent with the finding that *mec-7* and *mec-12* levels are limiting in the *mbl-1(0)* mutant. Please see details (line-428-437, Page-18-19)

(2) Whether mec-7 and mec-12 levels are altered by the klp-7 mutation, and whether these defects are again corrected by adding the mbl-1 mutation.

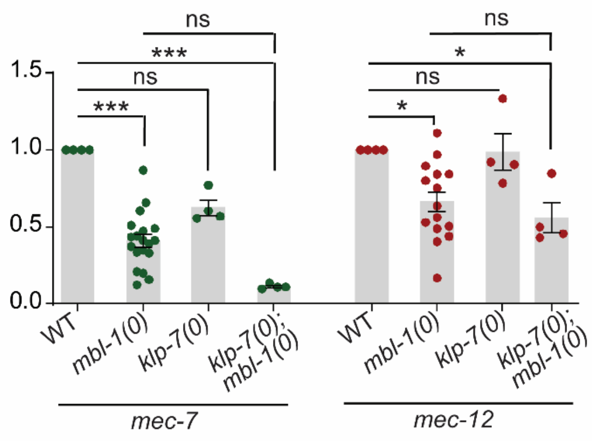

Response- The *mec-7* and *mec-12* levels in the *klp-7* mutant background are not significantly altered. Our interpretation is that KLP-7 is not involved in the transcriptional or post-translational regulation of *mec-7* or *mec-12*. This is consistent with the fact that KLP-7 is not a transcriptional regulator. Please see the panel above. We have done this experiment specifically to address this point.

3. What is the link between mec-7/mec-12 regulation by mbl-1 and intact microtubule polarity and dynamics in the touch neurons? As had been stated above, this is the most important mechanistic issue for the current study that needs to be rigorously explored by experiments.

Response- It has been shown by Martin Chalfie and others that *mec-12* and *mec-7* are the touch neuron-specific tubulins which are the building blocks of the characteristic fifteen protofilament microtubules in touch neurons. The *mec-12* and *mec-7* mutants have defects in

Telephone: +91-124-2845 237 Fax : +91-124-2338910
Email : anindya@nbrc.ac.in **Website : www.nbrc.ac.in**

microtubule architecture and neurite growth (Lee et al MBoC 2021, Zheng et al MBoC 2017, Lockhead et al MBoC 2016). These references are also available in the introduction, discussion section, and in the main text.

In a previous article from our lab, we have shown that increased microtubule instability (dynamics) leads to the formation of mixed polarity microtubules. Whereas increased stability in the microtubule supports uni-parallel plus end-out orientation of MTs in the anterior neurite of PLM (Puri et al 2021 JCB). Increased microtubule instability is linked to the mixed polarity of microtubules in the case of the posterior neurite of PLM (Puri et al 2021 JCB). Similarly, in vertebrate dendrites, the microtubules are relatively unstable as compared to the axon, and microtubules are arranged in random orientation (Witte et al 2008 JCB and Yau et al 2016; J Neuroscience).

In this report, we are showing that in *mbl-1* mutant microtubule instability is increased causing mixed-polarity organization of microtubules (Figure 3). In our view, a reduced amount of *mec-7* and *mec-12* tubulin-subunits in *mbl-1* mutant leads to decreased polymerization rate (or increased depolymerization) leading to increased dynamics of microtubules, which in turn causes mixed polarity in microtubules. We added the evidence that independently *mec-7* and *mec-12* mutants show the mixed-polarity organization of microtubules in the anterior neurites of PLM (Figure S7B-F). See the (line-428-437, Page-18-19) for details.

Also, we have clearly addressed these mechanistic insights in the discussion section. Please see the (line-536-570, P-23-24) for details.

4. What is the basis of axon defects in the D-type motor neurons of the mbl-1 mutant? Obviously, this is unlikely to be the consequence of altered mec-7 and mec-12 levels, as these two tubulin genes are not expressed in DD or VD neurons. Can the authors speculate from the candidate MBL-1 bound RNAs (Figure 4A) and elaborate on this issue (by discussion or experiments)?

Response- Thanks for the query. We agree with the reviewer that *mec-7* and *mec-12* do not express in D-type motor neurons. MBL-1 has a binding site on transcripts that are known to regulate axon growth like *unc-76*, *ced-10*, *mig-2*, *unc-73*, and *max-2*. There is a report which shows that *max-2* has an axon growth defect (Mark Lucanic et al 2006; Mahekta R. Gujar et al 2017; Laird Bloom and H. Robert Horvitz 1997).

5. Figure 5D, 5E: The interpretation is flawed that instability of mec-7 and mec-12 transcripts is increased in the mbl-1 mutant. Since the expression of these two genes is already low in the mbl-1 mutant, the authors need to normalize the levels of mec-7 and mec-12 transcripts after Actinomycin D to those at baseline, and compare this value to that of the wild type animals. Without such quantification, the current conclusion that mbl-1 promotes the stability of mec-7 and mec-12 transcripts is premature and unsupported.

Anindya Ghosh Roy, PhD
Associate Professor, Wellcome Trust-DBT Intermediate Fellow

National Brain Research Center (Deemed University)
(An Autonomous Institute of Deptt. of Biotechnology, Ministry of Science and Technology, Govt. of India)
NH-8, Nainwal Mode, Manesar-122051 (Haryana)

Please see the response to similar comments from reviewer-1.

We have made it clear now, please see the revised Figure 6 and the explanation on page 16-17, line 384-405 .

We agree that it is important to normalize the levels of *mec-7* and *mec-12* transcripts after Actinomycin D to those at baseline. We have compared *mec-7* and *mec-12* mRNA levels in the presence or absence of Act D in both WT and *mbl-1(0)* backgrounds. *mec7/ mec-12* transcript levels in the presence of Act D were normalized to *mec-7/mec-12* levels in the absence of Act D in both WT and *mbl-1(0)* mutants. In the WT background, there was a significant reduction in *mec-7/mec-12* transcript levels in the presence of Act D. However the *mbl-1(0)* mutants showed a further drop in *mec-7/mec-12* transcript levels upon Act D treatment. The fold reduction in *mec-7* transcript due to ActD treatment in wild-type vs *mbl-1* mutant is 0.7462 (25%) vs 0.4170 (58%). Similarly, the fold reduction in *mec-12* transcript in wild-type vs *mbl-1* mutant is 0.8245 (18%) vs 0.6689 (33%). And these are all statistically significant (see figure-6D-E, and its legend, also shown below is 6E) Therefore, we believe that the enhanced degradation of *mec-7/mec-12* transcripts detected in the *mbl-1(0)* mutant upon Act-D treatment is indicative of MBL-1 influencing the stability of these transcripts. Please also see the revised text for a better explanation (line-387-412, Page17-18)

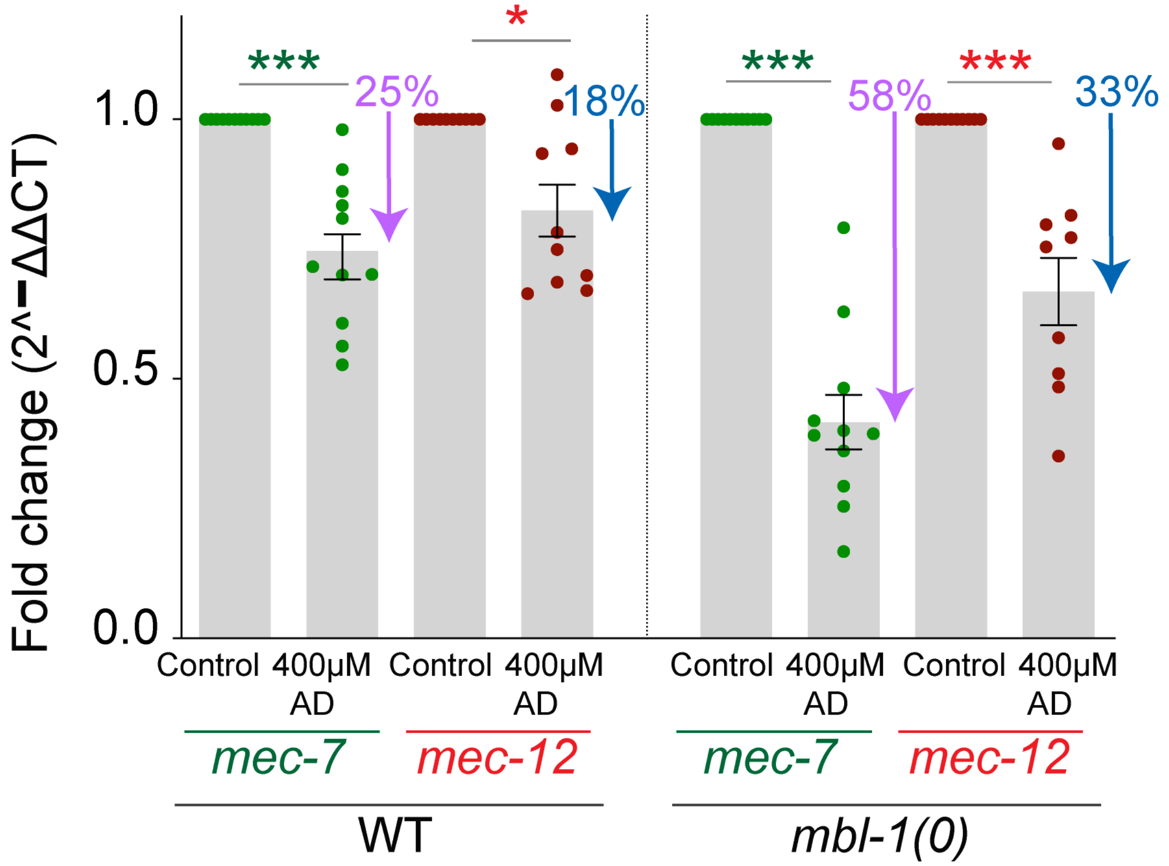

Telephone: +91-124-2845 237 Fax : +91-124-2338910
Email : anindya@nbrc.ac.in **Website : www.nbrc.ac.in**

Anindya Ghosh Roy, PhD
Associate Professor, Wellcome Trust-DBT Intermediate Fellow

National Brain Research Centre NBRC

National Brain Research Center (Deemed University)
(An Autonomous Institute of Deptt. of Biotechnology, Ministry of Science and Technology, Govt. of India)
NH-8, Nainwal Mode, Manesar-122051 (Haryana)

6. Figure 5F, 5G, Line 467-472: I could not find statements of using Actinomycin D in this experiment, so I assumed that it was conducted without Actinomycin D. If so, then the data presented here cannot be used to support the role of MBL-1 in maintaining MEC-7 stability. Moreover, the cytosolic mCherry protein was expressed from a different transgene using a different promoter (Pmec-4) from the mec-7-expressing transgene driven from the mec-7 promoter. As such, comparing the GFP to mCherry ratio using these two transgenes is not a valid way of normalizing MEC-7::GFP quantity. The authors need to create a Pmec-7::MEC-7::GFP::SL2::mCherry transgene for this purpose. That said, these experiments only explore the role of MBL-1 in controlling MEC-7 level without specifically examining its function in promoting MEC-7 stability, which clearly requires the use of Actinomycin D. These experiments need rigorous revision.

Response- Thanks for the suggestions. We have made the construct *Pmec-7*::MEC-7::GFP::SL2::MCherry as suggested by the reviewer. We reproduced the observation that MEC-7::GFP level as compared to MCherry is significantly dropped in *mbl-1* mutant with the suggested reagent (Figure S6 G-H, J). We have made a similar observation with the GFP::MEC-12 reporter suggesting that lack of *mbl-1* causes a reduction in both the tubulin protein levels in PLM neurons. And this is likely due to the drop in the transcript level of the *mec-7/12* as shown in Figure 6A-C.

We have also performed this imaging experiment with Act-D treatment. However, in the imaging experiments, we did not observe any additional decrease in MEC-7::GFP level in wild-type background due to ActD treatment. Similarly, we did not find a decrease in MEC-7::GFP level in *mbl-1* mutant due to ActD treatment. We believe that the perdurance of GFP is very high and it is already known that it is not sensitive enough to detect a smaller decrease in the transcript level (He et al 2019 eLife and Xianqiang Li et al 1998) as efficiently as quantitative RT-PCR. Therefore, our quantitative RT PCR experiment is a more relevant experiment to detect RNA stability-related changes.

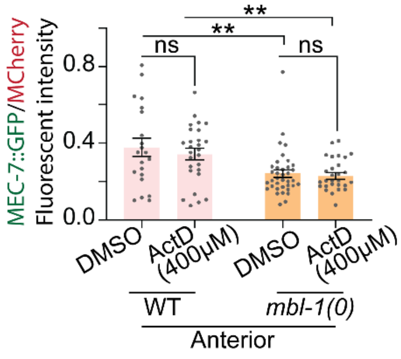

7. Figure 6: A negative control for the RNA-IP is necessary to show that MBL-1 does not bind most RNAs in a non-specific fashion. Using the KLP-7::GFP as a control is fine, but the authors need to show that not all RNAs are enriched in the RIP samples of MBL-1::GFP worms.

Anindya Ghosh Roy, PhD
Associate Professor, Wellcome Trust-DBT Intermediate Fellow

National Brain Research Centre NBRC

National Brain Research Center (Deemed University)
(An Autonomous Institute of Deptt. of Biotechnology, Ministry of Science and Technology, Govt. of India)
NH-8, Nainwal Mode, Manesar-122051 (Haryana)

Response- We have used *tba-1* as an internal control. In our qRT PCR, the CT value for *tba-1* gene was the same for the strains expressing P*mec-4*::GFP and P*mec-4*::MBL-1::GFP. Similarly, the CT value for the *tba-1* gene was the same from the immunoprecipitation used for *pklp-7::*KLP-7::GFP (control) and *pmbl-1::*MBL-1::GFP strains (IP sample).

Similarly, we did not observe any enrichment for *unc-76* (Figure 7D) in the pulldown from *pmbl-1::*MBL-1 strains (IP sample). These results suggest that MBL-1 is not binding to all the transcripts in a non-specific fashion. Please see the plot for unc-76 below.

unc-76 expression on FLAG IP

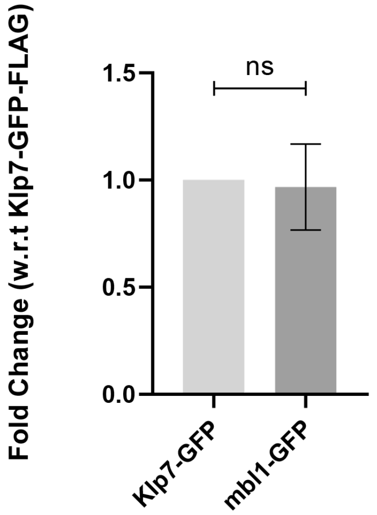

Minor points:

1. Line 47 and 107: please explain sad-1 (such as “sad-1/SAD BRSK kinase”)

Response- Thanks for suggesting. We have made the suggested change in both lines. Please see the (line-314-316, Page-14).

2. Figure 1B: It is better to just show the gene structure of mbl-1 and what the ju1128 and tm1563 alleles are, rather than showing the fosmid contents.

Response- We have included the gene structure of *mbl-1* with marked locations of mutations and we have removed other genes from the illustration. Please see the figure1B.

Anindya Ghosh Roy, PhD
Associate Professor, Wellcome Trust-DBT Intermediate Fellow

National Brain Research Center (Deemed University)
(An Autonomous Institute of Deptt. of Biotechnology, Ministry of Science and Technology, Govt. of India)
NH-8, Nainwal Mode, Manesar-122051 (Haryana)

3. The authors are suggested to provide high power images for the neurite and synaptic branch defects of the mbl-1 mutants as well as MBL-1::GFP in Figure 2. It is hard to see the details of the ALM and PLM phenotypes in the current images (Figures 2A, 2C, 2D and 2F).

Responses- We have thoroughly revised Figure-2 to highlight the synaptic branch defect in *mbl-1* mutant. Please see the revised version.

4. Line 122: The term "multiple axon-like phenotype" (of ALM) is a bit confusing. Although there is still debate whether this is a bipolar ALM phenotype or a misguidance defect of the ALM process, the term that the authors used does not help clarify the issue. I suggest that they just say "ectopic ALM process" (to be exact, the long process of ALM is not a pure axon – it displays features of dendrites).

Response- We have replaced the "Multiple axon-like phenotypes" with "ectopic neurite" in all the places For example, please see the revised title of the result section talking about the suppressor related to Figure-1(line-116-117, Page-6).

5. Line 182: Please correct this sentence: "making a ventral synaptic branch the PLM axon makes a synapse…"

Response- We have corrected the structural error. Please see page 6, line 121-123.

6. Line 192: "Pmec-4" should be italicized

Response- We have corrected and italicized.

7. Line 242: Gaps in the dorsal axons of D-type motor neurons should be interpreted as defects in axon extension similar to the short ALM and PLM neurites, rather than as synaptic defects. The authors should examine the distribution of RAB-3 and ELKS-1 in the D-type motor neurons of the mbl-1 mutant to substantiate their conclusion that mbl-1 is required for synaptic development in this class of neurons.

Response- Thanks for the suggestion. We do agree that the defect observed can be due to a defect in axon extension. We don't have any reagents to study the distribution of Rab-3 or ELKS-1 in the D-type motor neurons. So we have edited the conclusion as a defect in axon extension. Please see -lines 212-217, Page-10.

8. Line 246-247: Please annotate these genes succinctly.

Response- We have made the suggested changes. Please see lines 223-227, Page-10

9. Line 252: correct "caused due"

Response- We have changed it to 'caused by'. Please see the line 235, Page 11

Telephone: +91-124-2845 237 Fax : +91-124-2338910
Email : anindya@nbrc.ac.in **Website : www.nbrc.ac.in**

Anindya Ghosh Roy, PhD
Associate Professor, Wellcome Trust-DBT Intermediate Fellow

National Brain Research Center (Deemed University)
(An Autonomous Institute of Deptt. of Biotechnology, Ministry of Science and Technology, Govt. of India)
NH-8, Nainwal Mode, Manesar-122051 (Haryana)

10. Figure 3: The green and purple lines in embedded in Figure 3B are very hard to see. Figure 3J: Some lines are skewed.

Response- We have made the line clear in Figure 3B. The panel number for Figure 3J is now Figure 4G-H, we have made the line straight.

11. Line 263: "%" should be "percentage".

Response- We have changed % to 'percentage'.

12. Line 268: "smaller" should be "shorter".

Response- We have edited it to be shorter. Please see line 252 and 253, Page 11.

13. It is not clear what microtubule "arrangement" means in the paper. Microtubule "polarity" or "orientation" could be a better term, or the authors need to provide convincing justification for using such a term.

Response- Thanks for the query. We have edited it to 'microtubule polarity' or 'orientation'. In the first version of this article, we had used 'microtubule arrangement' to describe the polarity of microtubules in the neurites, either "plus-end-out" or "minus-end-out". We have taken care of this issue in the entire revised version of this manuscript

14. Line 382-387: I am confused here as the authors said that they found mec-7 and mec-12 were among genes found to be downregulated by mbl-1 in some published reports but claimed that they could not find genes in this group that were related to GO terms of axon development or synapse structure. Aren't mec-7 and mec-12 two such genes?

Response- Thanks for the query. *mec-7*, and *mec-12* were panned out from the GO term "MT-based process". For some reason, *mec-7* and *mec-12* did not belong to the 'axon growth' GO term. (Matilinen et al 2021 Genetics 10.1093/genetics/iyab114)

15. Line 420: The expression of "0.4717 +/- 0.03720 fold" is very strange and inappropriate. This could be seen again in Line 456. The authors should carefully revise their use of scientific expression. As has been stated above, this manuscript needs a thorough editing of writing. The senior author should take the responsibility to ensure the quality of writing.

Response- We have made the suggested changes. Please refer to lines 387-412, Pages 17-18.

16. Figure 6F, left: The protein band for MBL-1::GFP seems to be absent in the total lysate of the MBL-1::GFP::FLAG animals. The authors need to exlain this. A minor point is that the protein bands in the total lysates of the MBL-1::GFP::FLAG and Pmec-4::MBL-1::GFP look quite different. Why?

Telephone: +91-124-2845 237 Fax : +91-124-2338910
Email : anindya@nbrc.ac.in **Website : www.nbrc.ac.in**

Anindya Ghosh Roy, PhD
Associate Professor, Wellcome Trust-DBT Intermediate Fellow

National Brain Research Center (Deemed University)
(An Autonomous Institute of Deptt. of Biotechnology, Ministry of Science and Technology, Govt. of India)
NH-8, Nainwal Mode, Manesar-122051 (Haryana)

Response-Thanks for your query. Please see the revised panel with a revised western-blot (Figure 7B). MBL-1::GFP is visible in both total lysate and “FLAG-IP” sample.

The MBL-1::GFP::FLAG expresses all seven isoforms of MBL-1 as the endogenous MBL-1 is tagged with GFP::FLAG in this strain. Whereas in the *Pmec-4*::MBL-1::GFP transgenic construct, we have used the cDNA of C isoform of *mbl-1*. This could be a possible reason that there is a difference in protein band size/pattern in the total lysate of the MBL-1::GFP::FLAG and *Pmec-4*::MBL-1::GFP(C-isoform).

17. Figure 7A, the third image from top: That should be the AVM neuron, not the AVA neuron.

Response- Sorry for the error and thanks for pointing it out. We have corrected it. Please see the revised Figure 8 A.

Reviewer #3: Puri et al. reveal the role of mbl-1 in neurite and synapse biology through regulation of microtubule cytoskeleton components. In a genetic screen for suppressors of ectopically extended neurites in the ALM neuron in C. elegans, they identify a mutation in mbl-1 that shortens neurites in klp-7 mutants that have enhanced microtubule stability. mbl-1 mutants displayed mixed microtubule polarity and defects in axonal transport of cargo. MBL-1 is an RNA-binding protein, and the authors show evidence that it regulates the mRNAs of the microtubule genes mec-7 and mec-12, as well as sad-1. The authors propose that MBL-1 binds these mRNAs and stabilises them or mediates splicing in the case of sad-1. Overall, the manuscript is technically robust, novel, and interesting, however some of the mechanistic data into MBL-1 function needs improving as does the explanation of results that do not fit the conclusions made. There are some outstanding questions that also remain unaddressed.

Major:

1. The interpretation that MBL-1 stabilises the mRNA molecules of mec-7 and mec-12 is based on insufficient data. While the use of Actinomycin D is good, it may not block transcription fully and the authors need to prove whether this is indeed the case in their animals.

Response- Thanks for this comment. We have revised the analysis of how Actinomycin D treatment differentially affects the expression of *mbl-1* targets (e.g *mec-7*) in the wild type Vs *mbl-1* mutant background in revised Figure 6D-E. Please also see the revised text for a better explanation (line-387-412, Page17-18)

Also please see the response to the comments of the other two referees above to address this point.

The previous literature clearly has also demonstrated that Actinomycin D significantly blocks transcription in various model systems including the worm model. Here is the literature we cited in the manuscript for the use of ActinomycinD as a transcription inhibitor.

Telephone: +91-124-2845 237 Fax : +91-124-2338910
Email : anindya@nbrc.ac.in **Website : www.nbrc.ac.in**

Anindya Ghosh Roy, PhD
Associate Professor, Wellcome Trust-DBT Intermediate Fellow

National Brain Research Centre NBRC

National Brain Research Center (Deemed University)
(An Autonomous Institute of Deptt. of Biotechnology, Ministry of Science and Technology, Govt. of India)
NH-8, Nainwal Mode, Manesar-122051 (Haryana)

"63. Bensaude O. Inhibiting eukaryotic transcription: Which compound to choose? How to evaluate its activity? Transcription. 2011;2(3):103-8.

64. Lai WS, Arvola RM, Goldstrohm AC, Blackshear PJ. Inhibiting transcription in cultured metazoan cells with actinomycin D to monitor mRNA turnover. Methods. 2019;155:77-87.

65. Freytag V, Probst S, Hadziselimovic N, Boglari C, Hauser Y, Peter F, et al. Genome-Wide Temporal Expression Profiling in Caenorhabditis elegans Identifies a Core Gene Set Related to Long-Term Memory. J Neurosci. 2017;37(28):6661-72.

66. Wu J, Jiang X, Li Y, Zhu T, Zhang J, Zhang Z, et al. PHA-4/FoxA senses nucleolar stress to regulate lipid accumulation in Caenorhabditis elegans. Nat Commun. 2018;9(1):1195."

Additionally, see we have clearly demonstrated with quantitative RT-PCR that in the wild-type background both *mec-7* and *mec-12* levels are reduced significantly upon Actinomycin-D treatment (Figure 6D-E, Again see the E panel below). Moreover, please see that due to Actinomycin-D treatment in *mbl1-1* mutant, the quantitative drop in *mec-7/12* is higher. This is indicating the role of MBL-1 in the stability of these transcripts.

Telephone: +91-124-2845 237 Fax : +91-124-2338910
Email : anindya@nbrc.ac.in **Website : www.nbrc.ac.in**

Anindya Ghosh Roy, PhD
Associate Professor, Wellcome Trust-DBT Intermediate Fellow

National Brain Research Centre NBRC

National Brain Research Center (Deemed University)
(An Autonomous Institute of Deptt. of Biotechnology, Ministry of Science and Technology, Govt. of India)
NH-8, Nainwal Mode, Manesar-122051 (Haryana)

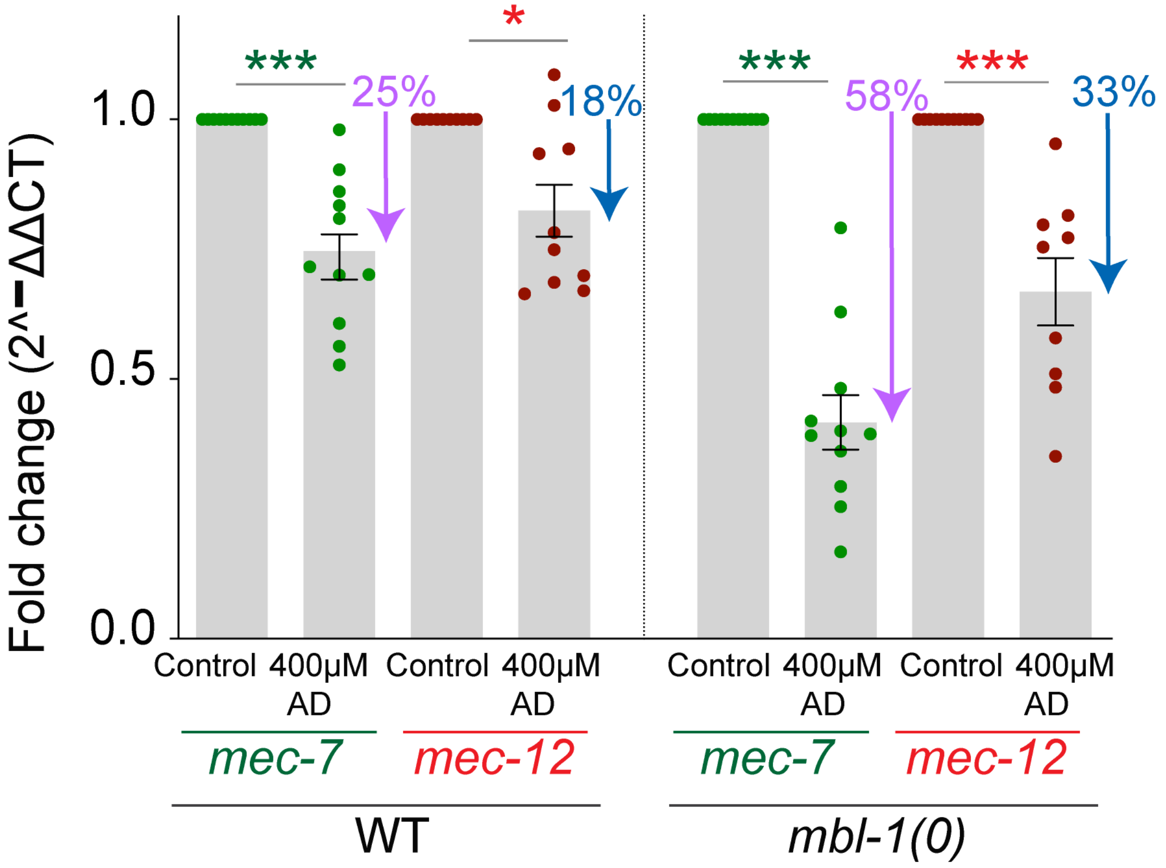

Moreover, the translational MEC-7::GFP reporter cannot distinguish between transcriptional or post-transcriptional mec-7 regulation. Therefore, other orthogonal evidence is required to conclude that MBL-1 stabilises these specific transcripts.

Response- Please see the Figure S6G-H panel. According to the suggestions made by reviewer-2, we have designed a reporter *Pmec-7*:: MEC-7::GFP::SL2::MCherry, that expresses both MEC-7::GFP and MCHerry under the same promoter. The expression of both MCherry (control) and MEC-7::GFP (experimental) reporters under the same promoter accounts for the transcriptional variation across the wild-type and *mbl-1* mutant genotypes. The MCherry level is not dropped in the *mbl-1* mutant (Figure S6I) indicating a specific decrease of MEC-7::GFP independent of its control under the *mec-4* promoter. This should likely be attributed to the translational reduction in MEC-7::GFP level in *mbl-1* mutant.

Moreover, we attempted to do the imaging experiments of the translational reporter (MEC-7::GFP) using the Actinomycin-D drug on the plate. we did not observe any additional decrease in MEC-7::GFP level in wild-type background due to ActD treatment. Similarly, we did not find a decrease in MEC-7::GFP level in *mbl-1* mutant due to ActD treatment. We believe that the

Anindya Ghosh Roy, PhD
Associate Professor, Wellcome Trust-DBT Intermediate Fellow

National Brain Research Center (Deemed University)
(An Autonomous Institute of Deptt. of Biotechnology, Ministry of Science and Technology, Govt. of India)
NH-8, Nainwal Mode, Manesar-122051 (Haryana)

perdurance of GFP is very high and it is already known that it is not sensitive enough to detect a smaller decrease in the transcript level (He et al 2019 eLife and Xianqiang Li et al 1998) as efficiently as quantitative RT-PCR. Therefore, our quantitative RT PCR experiment is a more relevant experiment to detect RNA stability-related changes.

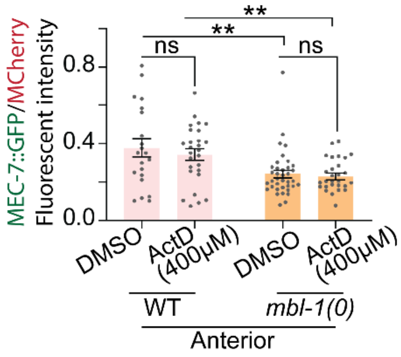

Finally, we understand that there are some shortcomings in understanding the mechanism by which MBL-1 acts on *mec-7* and *mec-12* mRNA. Our data is suggestive that it is through a stabilization mechanism (Figure 6E). However, one can not rule out other possibilities. The fact is that we have convincingly shown with multiple pieces of evidence that MBL-1 is responsible for maintaining the normal level of *mec-7* and *mec-12* in touch neurons.. i) qRT PCR, ii) immunoprecipitation and iii) imaging translational reporters. However, we are humble about the fact that there are unknowns in terms of the details. Other possibilities do not alter the main conclusion of the manuscripts that ‘MBL-1 regulates microtubule cytoskeleton in TRN through the *mec-7* and *mec-12* molecules.

2. Some of the data do not fit the conclusions. From the RIP experiments, MBL-1 associates with mec-7 and sad-1 mRNAs in mechanosensory neurons, but not mec-12. In light of this, how do you reconcile its regulation of mec-12 in PLM?

Telephone: +91-124-2845 237 Fax : +91-124-2338910
Email : anindya@nbrc.ac.in **Website : www.nbrc.ac.in**

Anindya Ghosh Roy, PhD
Associate Professor, Wellcome Trust-DBT Intermediate Fellow

National Brain Research Center (Deemed University)
(An Autonomous Institute of Deptt. of Biotechnology, Ministry of Science and Technology, Govt. of India)
NH-8, Nainwal Mode, Manesar-122051 (Haryana)

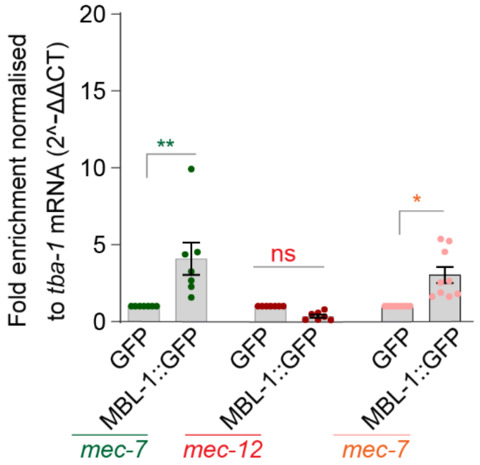

Response- We have done all of these experiments. Please see the results above. Although we successfully pulled down *mec-12* mRNA along with the *Pmbl-1*:FLAG::MBL-1::GFP. *mec-12* mRNA was not significantly enriched when we immunoprecipitated MBL-1::GFP(C isoform cDNA) from the transgenic strain expressing *Pmec-4*::MBL-1::GFP. A genomic copy of *mbl-1* expresses all possible isoforms, which most likely includes the isoform binding to *mec-12* mRNA. However, for MBL-1 pull down from mechanosensory neurons we used a single isoform (also our cDNA rescue construct) available to us (obtained from Yuji Kohara collection, Japan). No other isoform was available from Kohara collection and our effort to clone the other isoforms failed as of now. Therefore, we could not try this immunoprecipitation experiment with any other isoform yet. However, specific enrichment of *mec-12* in the immunoprecipitation with FLAG::MBL-1::GFP tells *mec-12* binds to MBL-1 as *mec-12* is mostly expressed in touch neuron.

Please see a more detailed explanation in response to reviewer-1.

See the (line-439-458, Page-19-20) for details. We also acknowledged and explained this in the discussion section (line-555-560, Page-24)

However, this does not alter the overall message of the manuscript that 'MBL-1 regulates microtubule cytoskeleton through the regulation of *mec-17* and *mec-12* mRNAs'. We clearly established that MBL-1 limits the expression of *mec-7* and *mec-12* and therefore this impacts microtubule dynamics in touch neuron.

Sad-1 mRNA levels or splicing are not altered in mbl-1(0) mutants, yet it associates with MBL-1 protein. If association results in mRNA stability or splicing, why aren't sad-1 mRNA levels lower or altered in length in animals lacking mbl-1?

Telephone: +91-124-2845 237 Fax : +91-124-2338910
Email : anindya@nbrc.ac.in Website : www.nbrc.ac.in

Anindya Ghosh Roy, PhD
Associate Professor, Wellcome Trust-DBT Intermediate Fellow

National Brain Research Center (Deemed University)
(An Autonomous Institute of Deptt. of Biotechnology, Ministry of Science and Technology, Govt. of India)
NH-8, Nainwal Mode, Manesar-122051 (Haryana)

Response- It is known from previous reports that SAD-1 has two isoforms and different cells express different isoforms. MBL-1 helps exclusion of 15th exon in the *sad-1* transcript. In the absence of *mbl-1,* an exon-included version of *sad-1* is produced in TRNs (Thompson et al 2019, eLife DOI: 10.7554/eLife.46726). We have found an association of SAD-1 transcript with MBL-1, but we did not find any change in transcript length. Since we used whole worm lysate, it might be difficult to find out the change in length because some cells express A isoform and some cells express B isoform. We have provided functional evidence that upon expressing the exon-included version of *sad-1* in TRN, the synapse defect could be rescued in *mbl-1(0).* However, the genomic version of *sad-1* couldn't rescue the synapse defect in the *mbl-1* mutant. This convincingly showed that synapse defect in *mbl-1* mutant involves the regulation of *sad-1* splicing through MBL-1.

We have also explained this in the discussion section of the revised manuscript (line-571-583, Page-24-25)

3. There are also some unanswered questions. Why do PLM anterior processes develop a mixed microtubule polarity if mec-7 and mec-12 mRNA levels are reduced in mbl-1(0) mutants? Why is the number of growing microtubules increased in mbl-1(0) mutants?

Response- Please see the response to similar comments from reviewer-2. It has been shown by Martin Chalfie and others that MEC-12 and MEC-7 are the touch neurons specific tubulins, which are required to make microtubule protofilaments (MARTIN CHALFIE and J . NICHOL THOMSON 1982, Alexander Bounoutas et al 2009). Here we have found that in the *mbl-1* mutant, tubulin (*mec-12* and *mec-7)* level is reduced and thereby reducing microtubule polymerization or increasing depolymerization. Consistent with this idea, we have also seen here that in loss of function mutants for *mec-7* and *mec-12*, the microtubule instability is increased as indicated by the EBP-2::GFP reporter assay (supporting figure-panel in Figure-S7B-F). Essentially, both in *mbl-1* and tubulin mutants (*mec-7/12*), the microtubule dynamics and polarity are affected in a similar manner.

Plus-end-binding reporter EBP-2::GFP reports the dynamic population of microtubules in cells (Ghosh-Roy et al 2012 Dev Cell, Stepanova T et al 2003, J Neurosci. 2003;23:2655–2664). In the field, this reporter is well-accepted in worms and other organisms. An increased number of growing microtubules as indicated in the analyzed kymographs represent increased microtubule instability or dynamics (Puri et al 2021 JCB, Ghosh-Roy et al 2012 Dev Cell, Stepanova T et al 2003, J Neurosci. 2003;23:2655–2664). Also, Increased microtubule instability is linked to the mixed polarity of microtubules in the case of the posterior process of PLM (Puri et al 2021, JCB). Similarly, in vertebrate dendrites, the microtubules are relatively unstable as compared to the axon, and microtubules are arranged in random orientation (Witte et al 2008 JCB and Yau et al 2016 J Neuroscience)

We have also explained this in the discussion section of the revised manuscript (line-562-565, Page-24)

[illegible]
Email : anindya@nbrc.ac.in Website : www.nbrc.ac.in

Anindya Ghosh Roy, PhD
Associate Professor, Wellcome Trust-DBT Intermediate Fellow

National Brain Research Center (Deemed University)
(An Autonomous Institute of Deptt. of Biotechnology, Ministry of Science and Technology, Govt. of India)
NH-8, Nainwal Mode, Manesar-122051 (Haryana)

MINOR.

At times, the manuscript is written in a descriptive manner (phenotypic data) and could be improved with more contextualisation and/or insight into the functional consequences of the observed neurite changes. Highlighting the conceptual advances or the point of each result throughout the manuscript may also improve readability in some sections.

Response- We appreciate your suggestion. We have revised the manuscript for better rationale and clarity.

Figure 2B: Authors should clarify in the main text and legend that TRN refers to mechanosensory neurons under pmec-4.

Response- Thanks for the suggestion. We have referred to the Figure legend.

Page 8 – line 183 – only photos for one of the mutant alleles is shown in fig. 2A.

Response- We have added images from both the alleles of *mbl-1* in Figure 2.

Page 8 – line 198 – The UNC-9::GFP reporter appears as puncta throughout the body of the wild-type animal in fig. S2D. Many of these puncta are missing in the mbl-1(0) mutant. Can the authors explain this? In addition, it is also very difficult to understand how the schematics in panel E of this figure were derived from the representative images. For example, there are many UNC-9::GFP puncta along the wild-type PLM axon that are not included in the schematic. What are these? This result needs better clarification.

Response- We have changed our illustration and in the results, we have explained these data. Please see revised Figure S2D-F.

Page 15 – line 351 – According to Table S3, 22 genes are noted are assessed, rather than 13.

Response- Thank you for pointing out the mistake. We have rectified it, Please see, lines 306-307, Page 13.

Page 21 –line 469 - missing mcherry transgene nomenclature.

Response- Thanks, we have made it *mCherry*. Please see the results section line 415, page 18.

Page 15 -lines 342 – 362 should be condensed. It reads as repetitious.

Response- Thanks for the suggestion, we tried rephrasing it. Please line 293-305, Pages 13.

Telephone: +91-124-2845 237 Fax : +91-124-2338910
Email : anindya@nbrc.ac.in **Website : www.nbrc.ac.in**

Anindya Ghosh Roy, PhD
Associate Professor, Wellcome Trust-DBT Intermediate Fellow

National Brain Research Center (Deemed University)
(An Autonomous Institute of Deptt. of Biotechnology, Ministry of Science and Technology, Govt. of India)
NH-8, Nainwal Mode, Manesar-122051 (Haryana)

Figure 4 - Authors should indicate what fluorescent reporter is used for the imaging and analysis. Figure needs images of controls/wild types for reference.

Response- We have added a control image labeled as ‘wild-type’ in Figure 5B panel. We have used the P*mec-7*::GFP (*muIs32*) reporter in this Figure, which is mentioned in the legend section for this figure, and also a written reporter which was used for imaging and analysis for other figures.

Some grammatical and sentence structure errors. Some parts of the text could be simplified to avoid confusion. The protein of interest is spelt incorrectly in the title.

Response- Thanks for pointing out the mistake and sorry for the mistake. We have rectified this. We have thoroughly revised the manuscript for language, grammar, and clarity.

Sincerely,

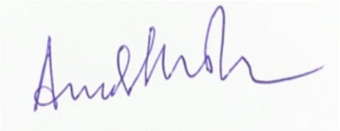

Anindya Ghosh Roy.

Telephone: +91-124-2845 237 Fax : +91-124-2338910
Email : anindya@nbrc.ac.in **Website : www.nbrc.ac.in**
