# Supplementary material for: Muscleblind-1 interacts with tubulin mRNAs to regulate the microtubule cytoskeleton in C. elegans mechanosensory neurons
Source: PLoS Genet. 2023 Aug 21;19(8):e1010885. doi: 10.1371/journal.pgen.1010885 (PMC10470942; doi:10.1371/journal.pgen.1010885)
Supplement: Attachment — Submitted filename: Response Letter-Puri etal-Revision2-120723.pdf [file pgen.1010885.s015.pdf]

Anindya Ghosh Roy, PhD
Associate Professor, Wellcome Trust-DBT Intermediate Fellow

National Brain Research Center (Deemed University)
(An Autonomous Institute of Deptt. of Biotechnology, Ministry of Science and Technology, Govt. of India)
NH-8, Nainwal Mode, Manesar-122051 (Haryana)

Point wise response

Dated, 12th July 2023, Gurgaon

Re: "Muscleblind-1 regulates microtubule cytoskeleton in C. elegans mechanosensory neuron through tubulin mRNAs" (PGENETICS-D-22-01039R1) by Puri et al.

Editor's note:

Thank you for submitting a revised version of your manuscript. The reviewers have examined the paper and they have found it highly improved compared to the original submission. However, they found that there are still some editing elements that needs to be addressed before the paper can proceed further. Importantly, they also highlighted that the manuscript still needs improvement in the writing, grammar, and sentences structure; please ensure that these also are addressed before your final submission.

Unless you believe additional time is needed, I trust a revised version can be submitted within 2 weeks.

Response: Thanks for the positive comments on the manuscript. We have made the suggested revisions and thoroughly edited the text to enhance the readability and coherence of the text. We have addressed all of the reviewers' comments. The section below contains a point by point response.

I look forward to receive your revised manuscript.

Thank you,

Kind regards,

Massimo.

Telephone: +91-124-2845 237 Fax : +91-124-2338910
Email : anindya@nbrc.ac.in **Website : www.nbrc.ac.in**

Anindya Ghosh Roy, PhD
Associate Professor, Wellcome Trust-DBT Intermediate Fellow

National Brain Research Center (Deemed University)
(An Autonomous Institute of Deptt. of Biotechnology, Ministry of Science and Technology, Govt. of India)
NH-8, Nainwal Mode, Manesar-122051 (Haryana)

Reviewer's Responses to Questions

Comments to the Authors:

Reviewer #1: The authors have addressed most of my previous concerns and the paper is now much improved from the first submission. However, I still have some minor concerns that should be addressed prior to the manuscript being published.

Response: Thanks for the positive comments on the revised manuscript.

1. The writing still needs significant improvement for clarity, and to correct grammatical errors and issues with sentence structure.

Response: We have thoroughly revised the manuscript for grammatical errors and other issues in the text.

2. The title should be revised for clarity and to better describe the study. I suggest the following: "Muscleblind-1 interacts with tubulin mRNAs to regulate the microtubule cytoskeleton in the C. elegans mechanosensory neurons"

Response: We have revised the title according to the suggestion.

3. The authors should provide a definition for the sad-1 gene in the abstract. Currently, no indication is given for what this gene is, and therefore there is no capacity for the reader to grasp the importance of the interaction between MBL-1 and sad-1.

Response: We have introduced and explained the sad-1 gene in the abstract. Please see page 2, line 35-38

4. The TRN phenotypes of the mbl-1 mutants are remarkably similar to those observed in the absence of MEC-17 (neuronal defects, MT defects, synaptic defects, interactions with MEC-12/MEC-7). Yet, there is no mention of MEC-17 in the manuscript. The authors should at least include a mention of the similarities and potential involvement of MEC-17 in their discussion.

Response: We have discussed the phenotypic similarity between mec-17 and mbl-1 mutants in the discussion section. We have referred Neumann B and Hilliard MA 2014 in the relevant discussion. Please see page 24, line 552-554.

Telephone: +91-124-2845 237 Fax : +91-124-2338910
Email : anindya@nbrc.ac.in **Website : www.nbrc.ac.in**

Anindya Ghosh Roy, PhD
Associate Professor, Wellcome Trust-DBT Intermediate Fellow

National Brain Research Centre NBRC

National Brain Research Center (Deemed University)
(An Autonomous Institute of Deptt. of Biotechnology, Ministry of Science and Technology, Govt. of India)
NH-8, Nainwal Mode, Manesar-122051 (Haryana)

Reviewer #2: The authors had done significant amount of work for the revision, and these additional data strengthened the conclusion and clarified most of the issues that I raised during the initial review of the paper. The revised manuscript was much better than the original one. I think the paper can now be considered for publication at PLoS Genetics, after the following minor points are addressed:

Response: Thanks for the positive comments on the revised manuscript.

1. sad-1 should be explained in the abstract.

Response: We have introduced and explained the sad-1 gene in the abstract. Please see page 2, line 35-38

2. In Author summary, the last statement "... might help mitigate the diseases such as muscular dystrophy caused due to misregulation of MBNL in humans" is better removed or tuned down. The study was conducted in neuronal cells, which have very different gene expression patterns and cell biology to those of the muscle cells. The current statement is misleading and exaggerating.

Response: We have revised the "Author summary" and the last sentence is now revised. Please see page 3, lines 60-62.

3. Line 119: The anterior cells should include AVM? Or the authors should just remove "anterior cells" and "posterior cells" to avoid confusion. As my comments in the initial review, the authors should avoid using jargons and instead stick with the standard terminology of the worm community.

Response: Thanks for this suggestion. We have removed the "anterior/posterior cells" jargon. Please see page 6, lines 124-126.

4. I cannot find description of the suppressor screen in the Materials & Methods section. A few descriptions will be necessary, such as the mutagenesis method (EMS?), an estimation of the screen size (number of haploid genomes screened), etc. By contrast, a detailed description of mapping ju1128 in the Results may not be necessary or should be streamlined (line 136-147).

Response: Thanks for this suggestion. We have added a section called "EMS Mutagenesis and Forward Genetic Screen Strategy" in the methods section. Please see page 26, lines 617-630.

Telephone: +91-124-2845 237 Fax : +91-124-2338910
Email : anindya@nbrc.ac.in **Website : www.nbrc.ac.in**

Anindya Ghosh Roy, PhD
Associate Professor, Wellcome Trust-DBT Intermediate Fellow

National Brain Research Center (Deemed University)
(An Autonomous Institute of Deptt. of Biotechnology, Ministry of Science and Technology, Govt. of India)
NH-8, Nainwal Mode, Manesar-122051 (Haryana)

5. Line 175: "mbl" should be italicized.

Response: Please see page 8, line 180 .

6. Line 205: It should read "In both mutant alleles of mbl-1"

Response: Please see page 10, line 212.

7. The y-axis scales of figure S3C and S3H should be mended as a full 100% scale.

Response: We have updated the plots. Please see figure S3C, S3F and S3H.

8. Figure S7E: Statistics should be carefully checked again, as the data of the WT and mec-7 or mec-12 mutants look quite similar at the first glance.

Response: We have reanalyzed the data carefully. Please see the graph below. There was an error in the label for significance difference between WT and mec-12 posterior (pst) neurite, which has been corrected in the plot in Figure S7E. Thank you for pointing out this error. However, the growth length in mec-7 Posterior is significantly different from WT ($p<0.001$), as shown in graph below.

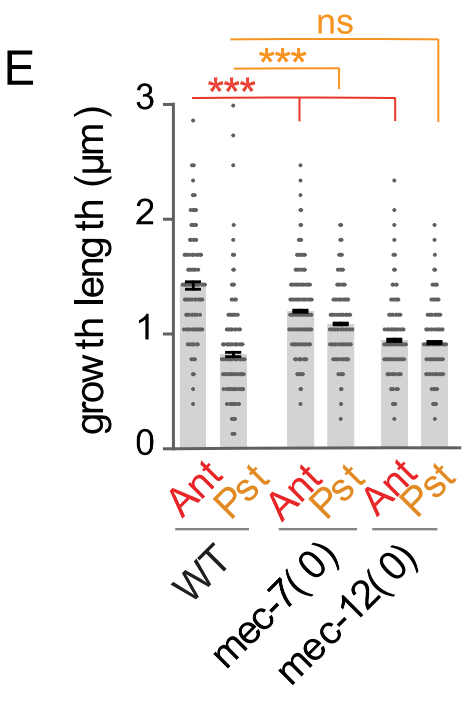

Telephone: +91-124-2845 237 Fax : +91-124-2338910
Email : anindya@nbrc.ac.in **Website : www.nbrc.ac.in**

Anindya Ghosh Roy, PhD
Associate Professor, Wellcome Trust-DBT Intermediate Fellow

National Brain Research Center
(Deemed University)
(An Autonomous Institute of Deptt. of Biotechnology,
Ministry of Science and Technology, Govt. of India)
NH-8, Nainwal Mode, Manesar-122051 (Haryana)

Reviewer #3: Puri et al have submitted a revised version of their manuscript. They have addressed all of my comments well and the manuscript is an improvement overall.

Response: We appreciate the positive comments on the revised manuscript. We have further revised the manuscript based on the comments of the other two reviewers.

Sincerely,

Anindya Ghosh Roy.

Telephone: +91-124-2845 237 Fax : +91-124-2338910
Email : anindya@nbrc.ac.in **Website : www.nbrc.ac.in**
